# Protocol for a MULTI-centre feasibility study to assess the use of ^99m^Tc-sestaMIBI SPECT/CT in the diagnosis of kidney tumours (MULTI-MIBI study)

**DOI:** 10.1136/bmjopen-2022-067496

**Published:** 2023-01-24

**Authors:** Hannah Warren, Thomas Wagner, Michael A Gorin, Steven Rowe, Beverley Fiona Holman, Deborah Pencharz, Soha El-Sheikh, Ravi Barod, Prasad Patki, Faiz Mumtaz, Axel Bex, Veeru Kasivisvanathan, Caroline M Moore, Nicholas Campain, Jon Cartledge, Andrew Scarsbrook, Fahim Hassan, Tim S O'Brien, Grant D Stewart, Iosif Mendichovszky, Sabina Dizdarevic, Ammar Alanbuki, William H Wildgoose, Tze Wah, Cecilia Vindrola-Padros, Elena Pizzo, Hakim-Moulay Dehbi, Paula Lorgelly, Kurinchi Gurusamy, Mark Emberton, Maxine G B Tran

**Affiliations:** 1 Division of Surgery and Interventional Science, University College London, London, UK; 2 Specialist Centre for Kidney Cancer, Royal Free Hospital, London, UK; 3 Department of Nuclear Medicine, Royal Free Hospital, London, UK; 4 Milton and Carroll Petrie Department of Urology, Icahn School of Medicine at Mount Sinai, New York, New York, USA; 5 Department of Urology, The James Buchanan Brady Urological Institute, Johns Hopkins University School of Medicine, Baltimore, MD, USA; 6 Russell H. Morgan Department of Radiology and Radiological Science, Johns Hopkins University School of Medicine, Baltimore, MD, USA; 7 Department of Pathology, Royal Free Hospital, London, UK; 8 Department of Urology, University College Hospital, London, UK; 9 Department of Urology, Royal Devon and Exeter NHS Foundation Trust, Exeter, UK; 10 Department of Urology, Leeds Teaching Hospitals NHS Trust, Leeds, UK; 11 Department of Radiology, Leeds Teaching Hospitals NHS Trust, Leeds, UK; 12 Department of Nuclear Medicine, Guy's and St Thomas' NHS Foundation Trust, London, UK; 13 Department of Urology, Guy's and St Thomas' NHS Foundation Trust, London, UK; 14 Department of Surgery, University of Cambridge, Cambridge, UK; 15 Department of Radiology, University of Cambridge, Cambridge, UK; 16 Department of Nuclear Medicine, Cambridge University Hospitals NHS Foundation Trust, Cambridge, UK; 17 Department of Nuclear Medicine, University Hospitals Sussex NHS Foundation Trust, Worthing, UK; 18 Brighton and Sussex Medical School, Brighton, UK; 19 Department of Urology, University Hospitals Sussex NHS Foundation Trust, Worthing, UK; 20 Patient Representative, London, UK; 21 Rapid Research, Evaluation and Appraisal Lab (RREAL), Department of Targeted Intervention, University College London, London, UK; 22 Department of Applied Health Research, University College London, London, UK; 23 Institute of Clinical Trials and Methodology, University College London, London, UK

**Keywords:** Urological tumours, Nuclear radiology, HEALTH ECONOMICS

## Abstract

**Introduction:**

The incidence of renal tumours is increasing and anatomic imaging cannot reliably distinguish benign tumours from renal cell carcinoma. Up to 30% of renal tumours are benign, with oncocytomas the most common type. Biopsy has not been routinely adopted in many centres due to concerns surrounding non-diagnostic rate, bleeding and tumour seeding. As a result, benign masses are often unnecessarily surgically resected. ^99m^Tc-sestamibi SPECT/CT has shown high diagnostic accuracy for benign renal oncocytomas and other oncocytic renal neoplasms of low malignant potential in single-centre studies. The primary aim of MULTI-MIBI is to assess feasibility of a multicentre study of ^99m^Tc-sestamibi SPECT/CT against a reference standard of histopathology from surgical resection or biopsy. Secondary aims of the study include obtaining estimates of ^99m^Tc-sestamibi SPECT/CT sensitivity and specificity and to inform the design and conduct of a future definitive trial.

**Methods and analysis:**

A feasibility prospective multicentre study of participants with indeterminate, clinical T1 renal tumours to undergo ^99m^Tc-sestamibi SPECT/CT (index test) compared with histopathology from biopsy or surgical resection (reference test). Interpretation of the index and reference tests will be blinded to the results of the other. Recruitment rate as well as estimates of sensitivity, specificity, positive and negative predictive value will be reported. Semistructured interviews with patients and clinicians will provide qualitative data to inform onward trial design and delivery. Training materials for ^99m^Tc-sestamibi SPECT/CT interpretation will be developed, assessed and optimised. Early health economic modelling using a decision analytic approach for different diagnostic strategies will be performed to understand the potential cost-effectiveness of ^99m^Tc-sestamibi SPECT/CT.

**Ethics and dissemination:**

Ethical approval has been granted (UK HRA REC 20/YH/0279) protocol V.5.0 dated 21/6/2022. Study outputs will be presented and published nationally and internationally.

**Trial registration number:**

ISRCTN12572202.

**Strengths and limitations of this study**

- MULTI-MIBI is the first multicentre prospective study to assess ^99m^Tc-sestamibi SPECT/CT in the evaluation of indeterminate renal tumours.
- A composite reference standard of biopsy or surgical pathology allows generalisability of results to patients unwilling or unable to undergo surgical resection.
- Blinding of clinicians interpreting index and reference tests reduces risk of bias.
- Possible study limitations include the risk of non-diagnostic renal tumour biopsies and tumour misclassification on biopsy.
- If the primary outcome (successful recruitment) is met, this will inform a large-scale multicentre study.

## Introduction

The widespread use of cross-sectional imaging has led to an increase in the incidental detection of renal tumours.^1^ Based on data from surgical series, it is estimated that up to 30% of renal tumours are benign,^2^ with an increasing prevalence of benign histology with decreasing tumour size.^3^ The most common type of benign tumour is the oncocytoma. Unlike renal cell carcinoma (RCC), which commonly requires treatment, renal oncocytomas can be safely managed expectantly.^4–6^ However, a critical challenge lies in the identification of benign renal tumours, as traditional anatomic imaging techniques such as ultrasound, CT and MRI are unable to reliably distinguish between the various renal tumour histologies. Although renal mass biopsy can help in this regards, the relatively high non-diagnostic rate (~15%) and associated risk of complications with this procedure have led to its limited adoption in clinical practice.^7 8^ Thus, the majority of patients presenting with an incidental renal mass undergo treatment for a presumed cancer, exposing those with benign tumours to unnecessary surgical risk while consuming significant health resources.^9^

Investigation of new imaging approaches to improve characterisation of incidentally detected small renal masses has been identified as a priority research need by the Renal Cancer Gap Analysis Collaborative, a group composed of clinicians, researchers, patients and caregivers.^10^ In recent years, ^99m^Tc-sestamibi SPECT/CT has emerged as a promising non-invasive tool for the identification of benign renal oncocytomas. ^99m^Tc-sestamibi is a lipophilic cationic radiopharmaceutical that readily accumulates in cells with high concentrations of mitochondria, such as renal oncocytomas.^11^ Conversely, most histologic subtypes of RCC are relatively devoid of mitochondria and express membrane multidrug resistance pumps, which are known to actively export ^99m^Tc-sestamibi out of cells.^11^ These biological differences result in oncocytomas appearing avid, or ‘hot’ and RCCs non-avid or ‘cold’ on MIBI-kidney studies. A systematic review and meta-analysis including 117 renal lesions from single-centre studies showed pooled sensitivity and specificity of MIBI-kidney to detect renal oncocytomas versus other renal lesions was 92% (95% CI 72% to 98%) and 88% (95% CI 79% to 94%), respectively.^12^ No previous trials of MIBI-kidney have been conducted in the United Kingdom (UK), and there have been no multicentre trials.

One potential limitation of ^99m^Tc-sestambi SPECT/CT imaging of renal tumours is that a subset of RCCs exhibits relatively high intracellular concentrations of mitochondria and, therefore, display uptake of the radiotracer.^13–15^ These tumours include the chromophobe subtype of RCC and other oncocytic/chromophobe RCC.^16^ It is reassuring to note that these tumours exhibit generally indolent behaviour and low metastatic potential with excellent outcomes on active surveillance.^17^ We, therefore, termed this group of tumours as oncocytic renal neoplasms of low malignant potential and suggest that with few exception identification of such cT1 tumours on ^99m^Tc-sestamibi SPECT/CT should be managed similarly to that of benign renal oncocytomas.

Given the excellent performance characteristics of ^99m^Tc-sestamibi SPECT/CT for the non-invasive identification of renal oncocytomas and oncocytic renal neoplasms of low malignant potential, there is interest in utilising this test within the UK National Health System (NHS). However, the literature on ^99m^Tc-sestamibi SPECT/CT remains limited to single centres reporting relatively few tumours. We have recently reported on a pump-priming pilot study in the UK.^18^ Herein, we present the protocol for our feasibility study with the following aims^1^: to evaluate the feasibility of a large scale, UK-based, multicentre, clinical trial of ^99m^Tc-sestamibi SPECT/CT in the diagnostic pathway for renal tumours and^2^ to obtain estimates of sensitivity and specificity with which to power a larger scale trial.

## Methods and analysis

Study methods are reported with reference to Standard Protocol Items: Recommendations for Interventional Trials Checklist (SPIRIT)^19^ and SPIRIT-Path extension for cellular and molecular pathology content in clinical trial protocols.^20^

### Study design

**Figure 1:**
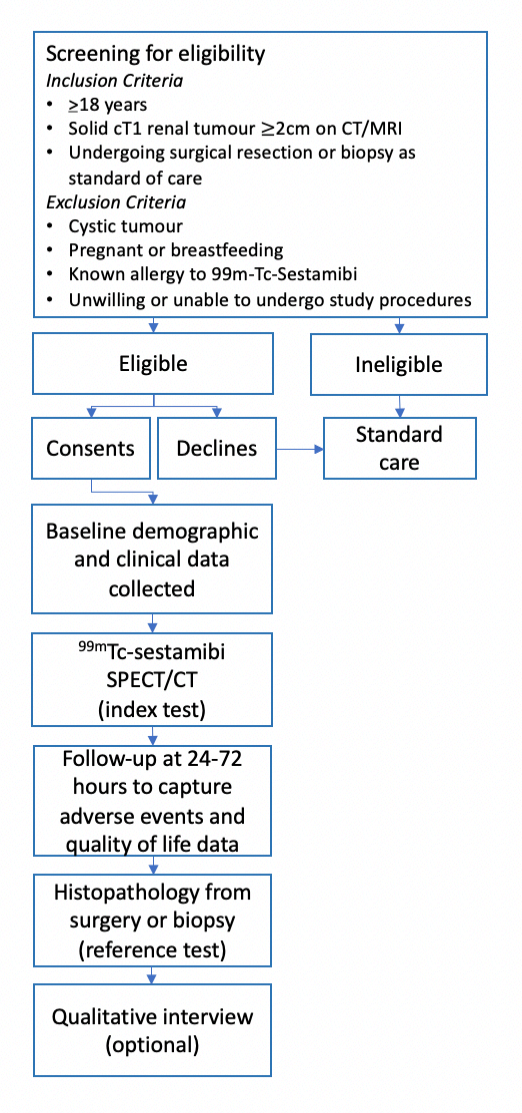
Study flow diagram.

A prospective, multicentre study to assess the feasibility and diagnostic performance characteristics of ^99m^TC-sestamibi SPECT/CT in adults (n=50) with solid, enhancing clinical renal tumours (2–7 cm) on cross-sectional imaging. The study design is summarised in figure 1.

### Objectives and outcomes

The primary aim of the study is to evaluate whether a multicentre diagnostic test evaluation study of ^99m^Tc-sestamibi SPECT/CT can recruit successfully. Secondary aims are to assess patient and clinician acceptability, refine inclusion/exclusion criteria, sample size requirements and determine clinician training needs for ^99m^Tc-sestamibi SPECT/CT interpretation.

The study objectives are to determine:

- Will patients consent to have a ^99m^Tc-sestamibi SPECT/CT prior to surgery or biopsy, including those from under-represented and underserved groups?
- What factors influence patient’s decisions to participate?
- What are the perceptions of clinicians and patients of ^99m^Tc-sestamibi SPECT/CT?
- What barriers and facilitators are there for adoption of ^99m^Tc-sestamibi SPECT/CT?
- What is the potential cost-effectiveness of using ^99m^Tc-sestamibi SPECT/CT within the NHS?
- What are the minimally acceptable criteria (MAC) for the sensitivity and specificity of ^99m^Tc-sestamibi SPECT/CT?
- Is it feasible to train nuclear medicine clinicians across the UK, including those serving under-represented and underserved communities, to interpret ^99m^Tc-sestamibi SPECT/CT?

The study outcomes are as follows:

Primary outcome

- Recruitment rate.

Secondary outcomes

- Sensitivity and specificity of MIBI to detect benign lesions in this study.
- Define the MAC for MIBI-kidney to be adopted in clinical practice, to inform the design and parameters of the future definitive clinical trial.
- Interobserver variability and training requirements in the interpretation of MIBI-kidney (local and central reports will be compared).
- Patient and clinician perceptions of utility and experience of MIBI-kidney scans and training.
- The evidence requirements for a cost-effectiveness analysis.

### Study setting

The study will be conducted in 3–6 NHS hospitals in England.

### Eligibility criteria

Consecutive patients discussed at specialist multidisciplinary team meetings will be screened for eligibility over a planned 15-month recruitment period. The inclusion criteria for entry to the study are adult patients (≥18 years) of any gender with a clinical T1 indeterminate solid renal tumour (2–7 cm) on cross-sectional imaging, willing and able to provide informed consent. Patients will be required to have surgery or renal tumour biopsy planned as part of their standard clinical care. Patients entering watchful waiting or active surveillance pathways without histologic diagnosis will be excluded. Other exclusion criteria will include cystic tumours, pregnant and breastfeeding patients, those with a known allergy to ^99m^Tc-sestamibi and those unwilling or unable to undergo the study procedures.

### Test methods

#### Index test

Nuclear medicine clinicians involved in the study will receive study-specific training on the interpretation of ^99m^Tc-sestamibi SPECT/CT from international experts at the beginning of the recruitment period. The training will include a lecture on ^99m^Tc-sestamibi SPECT/CT principles, ‘hands-on training’ supported by experienced faculty and a precourse and postcourse assessment.

900 MBq of ^99m^Tc-sestamibi will be injected intravenously in a single bolus, 75 min before SPECT/CT acquisition of the abdomen with the superior extent of the field-of-view set to the top of the liver dome. CT and SPECT image acquisition will follow manufacturer instructions and local experience. At minimum, we suggest that participating centres have SPECT/CT systems with the following specifications: at least two-slice helical diagnostic CT scanner, available low-energy all-purpose or low-energy high-resolution collimator, gamma camera or digital detector elements appropriate for 140-kEv photopeak acquisition and manufacturer-derived iterative reconstruction that includes scatter and attenuation correction.

The reporting clinician will document a qualitative assessment of the tumour as avid, non-avid or indeterminate on reconstructed SPECT/CT images, blinded to clinical information and the result of the histopathology reference test. A spherical region of interest will be drawn to measure maximum uptake in attenuation-corrected images within (a) the tumour and (b) the ipsilateral renal parenchyma. A ratio of maximum uptake between the tumour and normal renal parenchyma will be calculated. All ^99m^TC-sestamibi SPECT/CT scans will be transferred for central review at the lead site (Royal Free Hospital) and discordant reports resolved by discussion and consensus. Local site clinicians will report a subset of studies a second time at the end of the recruitment period to allow assessment of intrarater reliability.

#### Reference test

Histopathology from the final surgical resection specimen is considered the ‘gold standard’ diagnostic test to determine renal tumour subtype. It is worth noting that although biopsy allows for histological diagnosis, questions remain about the accuracy of this technique for determining the precise histology of a renal tumour, mostly relating to an approximately 15% non-diagnostic rate of this procedure^7 8^ and the need for architectural findings in the tissue sample to definitively diagnose some tumour types.^21^ Despite this, we feel a composite reference standard of surgery and biopsy allows generalisability of the results to non-surgical populations. To maximise the accuracy of this procedure, tumour biopsy will be performed using an image-guided approach by an interventional radiologist experienced in the technique. In the case of a non-diagnostic biopsy, the patient will be offered a second attempt, according to local guidelines.

Histopathological reporting of both biopsy and surgical samples will be performed by qualified pathologists at collaborating sites in accordance with the current WHO classification system for renal tumours,^16^ as per standard care. Pathologists will be blinded to the ^99m^Tc-sestamibi SPECT/CT result. Pathology slides/images will be exported for central review by a specialist uro-oncology pathologist and archiving at the lead site (Royal Free Hospital).

### Sample size and recruitment

The aim of this study is to assess the feasibility of a multicentre study of ^99m^TC-sestamibi SPECT/CT in the diagnostic pathway for renal tumours. Data from the feasibility phase will be used to inform the design and sample size of the definitive trial. We will aim to recruit 50 patients from 3–6 centres. This sample size will allow us to assess if 80% (95% CI 70% to 90%) of approached patients agree to undergo the study scan. Additionally, this sample size will have sufficient power to detect if there is a significant difference in the estimates of sensitivity between our study population and those reported in the literature. A sample size of 40 patients would achieve 81% power to detect a sensitivity of 0.65 (representing an estimate outside the lower end of the 95% CI for sensitivity from the literature) using a two-sided binomial test at the 5% two-sided alpha level. A 20% inflation to 50 patients, will allow for possible dropouts and other methodological challenges.

### Analysis

#### Qualitative study of feasibility and acceptability

Qualitative data obtained from semistructured interviews (conducted either by telephone or on virtual platforms for example, Microsoft Teams) with patients, carers and staff will be combined with documentary analysis (reports, meeting minutes) and will be used to inform within trial decision-making processes via a rapid feedback evaluation approach.^22^ Transcripts and key documents will be imported into NVivo and analysed using framework analysis.^23^ Data collection and analysis will be carried out in parallel and emerging findings will be shared with the trial team on a monthly basis to inform trial design and delivery.

The findings from the interviews and documentary analysis will be used to develop a discrete choice experiment to gain an understanding of preferences for trial participation and how participants trade-off different attributes of ^99m^TC-sestamibi SPECT/CT with other management scenarios. In addition, a survey will be conducted—informed by a rapid review of survey instruments reported in the published literature to capture the acceptability of interventions in clinical trials, to provide insights into the barriers or facilitators to patient decision-making and determine the degree of acceptability of ^99m^TC-sestamibi SPECT/CT.

#### Study of diagnostic accuracy

Diagnostic accuracy of ^99m^Tc-sestamibi SPECT/CT will be estimated by generating 2×2 tables for both avid and non-avid qualitative assessment, and relative radiotracer uptake ratio >0.6 and ≤0.6 for external validation of a predefined threshold from the literature.^24^ Analysis of a range of relative uptake ratios will be explored to assess performance at different thresholds. Diagnostic accuracy of ^99m^TC-sestamibi SPECT/CT will be calculated in terms of sensitivity, specificity and predictive values along with their 95% CIs. The prevalence of renal oncocytoma and other histology subtypes will be calculated with a 95% CI.

Inconclusive test results will be reported.^25^ The proportion of participants with invalid ^99m^TC-sestamibi SPECT/CT results, for example, due to technical failure, will be reported. The proportion of valid but inconclusive results will also be reported, and their impact on estimates will be assessed by including them as either test positive or test negative in sensitivity analyses. This is to inform how ^99m^Tc-sestamibi SPECT/CT might be used in the diagnostic pathway. If intended as a replacement test for histopathology, a valid but indeterminate ^99m^Tc-sestamibi SPECT/CT would be considered non-avid to avoid misclassifying malignant tumours as benign. If, however, ^99m^Tc-sestamibi SPECT/CT were to be used as a triage test, where avid tumours undergo confirmatory biopsy, then an indeterminate test could be considered avid to reduce the risk of surgery for benign pathology. The proportion of patients who do not complete the study schedule defined in the protocol will be calculated.

We will assess inter-rater and intrarater agreement using percentage agreement and Gwet’s first-order agreement coefficient.^26^

We do not anticipate the need to adjust for diagnostic drift for the reference test, given the short study duration. However, if current pathologic guidelines for renal neoplasia are updated during the course of the study archived samples will be rereviewed and reported according to the latest guidelines.

#### Study of health economics

Health economic modelling will be used to understand the potential cost-effectiveness of ^99m^TC-sestamibi SPECT/CT in the evaluation of patients presenting with an indeterminate renal mass.^27^ A decision analytic approach will compare the following scenarios:

1. Patients have empiric surgery (current standard-of-care).
2. Patients undergo tumour biopsy, those consistent with cancer have surgery and those with benign histology have active surveillance.
3. Patients undergo ^99m^Tc-sestamibi SPECT/CT, those with a ‘cold’ scan (suggestive of cancer) have surgery and those with a ‘hot’ scan (suggestive of benign tumour) have active surveillance.
4. Patients undergo ^99m^Tc-sestamibi SPECT/CT, those with a ‘cold’ scan have surgery, and those with a ‘hot’ scan have a confirmatory biopsy (MIBI would be likened to a triage test to select patients for biopsy for tissue confirmation before embarking on active surveillance).

The model will be populated with evidence from trial and published literature.^28^ Where data are not available, an expert elicitation approach will be employed to provide parameter values.^29^ The analysis will then compare the different approaches to standard-of-care by estimating the incremental cost-effectiveness ratios and assessing the uncertainty of these estimates using value of information (VOI) analysis. The VOI analysis will quantify the potential value of further research, identify areas of study with the greatest potential benefit and generate recommendations on future study designs.

### Data collection

Case report forms (CRFs) in paper and electronic format will be trialled. The CRFs will not bear the participant’s name or other directly identifiable data. The participants’ study ID will be used for identification purposes. Study-related procedures will be carried out during the baseline routine clinical visit, ^99m^Tc-sestamibi SPECT/CT visit and thereafter by telephone or email according to participant preference, as shown in table 1. CCRFs will be checked for completeness and accuracy by designated individuals against source data. Study data captured in paper format will be transcribed to an electronic database. Quality of life data will be captured using the previously validated EQ-5D-5L instrument.^30^ No analysis will begin until accuracy of the data has been assured. The final trial data set will be accessible to the chief investigator, statistician and health economist.

**Table 1:** Visit schedule and assessments

Procedures	Screening	Baseline	Intervention	24–72 hour follow-up	Follow-up (standard of care)	Interview follow-up
Demographics		X				
Medical history	X	X				
Consent (obtained by clinician/research nurse)		X				
Imaging	X					
^99m^Tc-sestamibi SPECT/CT			X			
QoL questionnaire		X		X		
Adverse event reporting			X	X		
Histology test and result					X	
Semi-structured interview						X

SPECT: Single photon emission computed tomography.

QoL: Quality of life.

A participant may withdraw their consent to participate at any time prior to the ^99m^Tc-sestamibi SPECT/CT scan. The decision to withdraw will be recorded in the CRF and medical notes. Participants withdrawing prior to ^99m^Tc-sestamibi SPECT/CT will be replaced. If following ^99m^Tc-sestamibi SPECT/CT, the participant states they do not wish to participate in scheduled follow-up (EQ-5D-5L completion), or deviate from the protocol, then data already collected will be kept and analysed. These patients will not be replaced.

Baseline data items will include the following:

- Baseline demographics (age, gender, ethnicity, medical and surgical history, current medication and allergies).
- Baseline blood test results (full blood count, renal function, coagulation screen).
- Baseline imaging (multiphase (to include non-contrast, arterial-phase, venous-phase and delayed-phase), contrast-enhanced CT or MRI of the abdomen).
- Renal tumour characteristics (complexity scoring, location, number of lesions).
- Quality of life questionnaire (EQ-5D-5L).

The following data on resource use will be collected at the time of the intervention

- Duration of visit to nuclear medicine department.
- Adverse events (AEs) during and immediately post-MIBI-kidney.

The following data will be collected at postintervention follow-up by telephone or email

- AEs following MIBI-kidney.
- Quality of life questionnaire (EQ-5D-5L).

After participation in the trial participants will continue follow-up as per standard care.

### Patient and public involvement

Patient and public involvement (PPI) has been central to the project concept and design. A prestudy PPI focus group informed the trial protocol and plain English summary. An online PPI survey received 231 responses and indicated 90% would be willing to participate in the proposed study. In addition to the qualitative workstream, PPI representatives from Kidney Cancer UK will form a study support group, meeting at regular intervals throughout the trial to provide advice and input on any trial challenges and developing/approving dissemination materials.

### Harms

^99m^Tc-Sestamibi has been used for cardiac and parathyroid imaging globally for decades and is known to be a safe radiopharmaceutical. The radiation exposure from one MIBI-kidney scan is 14 mSv, equivalent to approximately 5 years of average UK background radiation.^31^ As MIBI-kidney is the only study intervention in addition to standard care, a data-monitoring committee will not be required.

All AEs, whether related or unrelated to MIBI-kidney, will be documented in the patient’s notes, study CRF and the AE log. The AE log will be sent to the Sponsor (University College London & University College London Hospitals Joint Research Office) at least once per year. Incidental clinically significant abnormalities identified on MIBI-kidney will be recorded as AEs and communicated to the referring clinician and patient. All serious AEs will be recorded on an SAE form and reported to the Sponsor and relevant REC within 15 working days of the chief investigator becoming aware of the event.

### Auditing

Investigators and sites will permit trial-related monitoring, audit, REC review and regulatory inspection(s) and provide access to required data and documents.

### Ethics and dissemination

Ethical approval for this study has been granted (UK HRA REC 20/YH/0279). Protocol amendments will be promptly disseminated to Sponsor, investigators and trial steering committee members. The study is recorded on the trial registration website. The trial involves the administration of unsealed radioactive substances. An Administration of Radioactive Substances Advisory Committee certificate has been granted (AA-3990).

Study outputs will be presented at national and international conferences and published in peer-reviewed journals. Patient representatives will be involved in output dissemination to the public individual trial participants via study newsletter.

## Supplementary Material

Reviewer comments

Author's manuscript
