# Supplementary material for: Protocol for a MULTI-centre feasibility study to assess the use of 99mTc-sestaMIBI SPECT/CT in the diagnosis of kidney tumours (MULTI-MIBI study)
Source: BMJ Open. 2023 Jan 24;13(1):e067496. doi: 10.1136/bmjopen-2022-067496 (PMC9884914; doi:10.1136/bmjopen-2022-067496)
Supplement: Author's manuscript [file bmjopen-2022-067496.draft_revisions.pdf]

BMJ Open is committed to open peer review. As part of this commitment we make the peer review history of every article we publish publicly available.

When an article is published we post the peer reviewers' comments and the authors' responses online. We also post the versions of the paper that were used during peer review. These are the versions that the peer review comments apply to.

The versions of the paper that follow are the versions that were submitted during the peer review process. They are not the versions of record or the final published versions. They should not be cited or distributed as the published version of this manuscript.

BMJ Open is an open access journal and the full, final, typeset and author-corrected version of record of the manuscript is available on our site with no access controls, subscription charges or pay-per-view fees (http://bmjopen.bmj.com).

If you have any questions on BMJ Open's open peer review process please email info.bmjopen@bmj.com

BMJ Open

Protocol for a MULTI-centre feasibility study to assess the use of 99m Tc-sestaMIBI SPECT/CT in the diagnosis of kidney tumours (MULTI-MIBI study)

Journal:	*BMJ Open*
Manuscript ID	bmjopen-2022-067496
Article Type:	Protocol
Date Submitted by the Author:	16-Aug-2022

Complete List of Authors:	Warren, Hannah; University College London Division of Surgery and Interventional Science,<br>Wagner, Thomas; Royal Free Hospital, Department of Nuclear Medicine<br>Gorin, Michael; Icahn School of Medicine at Mount Sinai, Milton and Carroll Petrie Department of Urology<br>Rowe, Steven; Johns Hopkins University Department of Medicine, Radiology and Radiological Science<br>Holman, Beverley; Royal Free Hospital, Department of Nuclear Medicine<br>Pencharz, Deborah; Royal Free Hospital, Department of Nuclear Medicine<br>El-Sheikh, Soha; Royal Free Hospital, Department of Pathology<br>Barod, Ravi; Royal Free London NHS Foundation Trust, Specialist Centre for Kidney Cancer; University College London, Division of Surgery and Interventional Science<br>Patki, Prasad; Royal Free London NHS Foundation Trust, Specialist Centre for Kidney Cancer<br>Mumtaz, Faiz; Royal Free London NHS Foundation Trust, Specialist Centre for Kidney Cancer<br>Bex, Axel; University College London, Division of Surgery and Interventional Science; Royal Free London NHS Foundation Trust, Specialist Centre for Kidney Cancer<br>Kasivisvanathan, Veeru; University College London, Division Of Surgery and Interventional Science<br>Moore, Caroline; University College London, Division of Surgery and Interventional Science<br>Campain, Nicholas; Royal Devon and Exeter NHS Foundation Trust, Department of Urology<br>Cartledge, Jon; Leeds Teaching Hospitals NHS Trust, Department of Urology<br>Scarsbrook, Andrew; Leeds Teaching Hospitals NHS Trust, Department of Radiology<br>Hassan, Fahim; Guy's and St Thomas' NHS Foundation Trust, Department of Nuclear Medicine<br>O'Brien, Tim; Guy's and St Thomas' NHS Foundation Trust, Department of Urology<br>Stewart, Grant; University of Cambridge, Department of Surgery<br>Mendichovszky, Iosif; University of Cambridge, Department of Radiology; Cambridge University Hospitals NHS Foundation Trust, Department of Nuclear Medicine<br>Dizdarevic, Sabina; University Hospitals Sussex NHS Foundation Trust,

	Department of Nuclear Medicine; Brighton and Sussex Medical School<br>Alanbuki, Ammar; University Hospitals Sussex NHS Foundation Trust, Department of Urology<br>Wildgoose, William; Patient Representative<br>Wah, Tze; Leeds Teaching Hospitals NHS Trust, Department of Radiology<br>Vindrola-Padros, Cecilia; University College London, Rapid Research, Evaluation and Appraisal Lab (RREAL), Department of Targeted Intervention<br>Pizzo, Elena; University College London, Department of Applied Health Research<br>Dehbi, Hakim-Moulay; University College London Institute of Clinical Trials and Methodology<br>Lorgelly, Paula; University College London, Department of Applied Health Research<br>Gurusamy, Kurinchi; University College London, Department of Surgery and Interventional Science<br>Emberton, Mark; University College London, Division of Surgery and Interventional Science; University College Hospital, Department of Urology<br>Tran, Maxine ; University College London, Division of Surgery and Interventional Science; Royal Free Hospital, Specialist Centre for Kidney Cancer
Keywords:	Urological tumours < UROLOGY, Nuclear radiology < RADIOLOGY & IMAGING, HEALTH ECONOMICS

SCHOLARONE™
Manuscripts

TITLE PAGE

Title: Protocol for a MULTI-centre feasibility study to assess the use of ^{99m} Tc-sestaMIBI SPECT/CT in the diagnosis of kidney tumours (MULTI-MIBI study)

H Warren[1,2], T Wagner[3], MA Gorin[4], SP Rowe[5], BF Holman[3], D Pencharz[3], S El-Sheikh[2], R Barod[1,2], P Patki[2], F Mumtaz[2], A Bex[2], V Kasivisvanathan[1], C Moore[1], N Campain[6], J Cartledge[7], A Scarsbrook[8], F Hassan[9], TS O'Brien[10], GD Stewart[11], IA Mendichovszky[12,13], S Dizdarevic[14,15], A Alanbuki[16], W Wildgoose[17], TM Wah[8], C Vindrola-Padros[18], E Pizzo[19], HM Dehbi[20], P Lorgelly[18]9 K Gurusamy[1], M Emberton[1] and MGB Tran[1,2]

Corresponding author:
Hannah Warren[1,2]
[1]Division of Surgery and Interventional Science, University College London, UK
[2]Specialist Centre for Kidney Cancer, Royal Free Hospital, London, UK
h.warren@ucl.ac.uk

Affiliations:
[1]Division of Surgery and Interventional Science, University College London, UK
[2]Specialist Centre for Kidney Cancer, Royal Free Hospital, London, UK
[3]Department of Nuclear Medicine, Royal Free Hospital, London, UK
[4] Milton and Carroll Petrie Department of Urology, Icahn School of Medicine at Mount Sinai, New York, NY, USA
[5]Division of Nuclear Medicine and Molecular Imaging, The Russell H. Morgan Department of Radiology and Radiological Sciences, Johns Hopkins University School of Medicine, Baltimore, MD, USA
[6]Department of Urology Royal Devon & Exeter NHS Foundation Trust, UK
[7]Department of Urology, Leeds Teaching Hospitals NHS Trust, Leeds, UK
[8]Department of Radiology, Leeds Teaching Hospitals NHS Trust, Leeds, UK
[9]Department of Nuclear Medicine, Guy's & St Thomas' NHS Foundation Trust, London, UK
[10]Department of Urology, Guy's & St Thomas' NHS Foundation Trust, London, UK
[11]Department of Surgery, University of Cambridge, Cambridge Biomedical Campus, Cambridge, UK
[12]Department of Nuclear Medicine, Cambridge University Hospitals NHS Foundation Trust
[13]Department of Radiology, University of Cambridge
[14]Department of Nuclear Medicine, University Hospitals Sussex NHS Foundation Trust, UK
[15]Brighton and Sussex Medical School, UK
[16]Department of Urology, University Hospitals Sussex NHS Foundation Trust, UK
[17]Patient Advisor
[18]Rapid Research, Evaluation and Appraisal Lab (RREAL), Department of Targeted Intervention, University College London, UK
[19]Department of Applied Health Research, University College London, London, UK
[20]Comprehensive Clinical Trials Unit, Institute of Clinical Trials and Methodology, UCL, London, UK

For peer review only

ABSTRACT

Introduction

The incidence of renal tumours is increasing and anatomic imaging cannot reliably distinguish benign tumours from renal cell carcinoma (RCC). Up to 30% of renal tumours are benign, with oncocytomas the most common type. Biopsy has not been routinely adopted in many centres due to concerns surrounding non-diagnostic rate, bleeding, and tumour seeding. As a result, benign masses are often unnecessarily surgically resected. ^{99m}Tc-sestamibi SPECT/CT has shown high diagnostic accuracy for benign renal oncocytomas and other oncocytic renal neoplasms of low malignant potential in single-centre studies. The primary aim of MULTI-MIBI is to assess feasibility of a multi-centre study of ^{99m}Tc-sestamibi SPECT/CT against a reference standard of histopathology from surgical resection or biopsy. Secondary aims of the study include obtaining estimates of ^{99m}Tc-sestamibi SPECT/CT sensitivity and specificity and to inform the design and conduct of a future definitive trial.

Methods and analysis

A feasibility prospective multi-centre study of participants with indeterminate, clinical T1 renal tumours to undergo ^{99m}Tc-sestamibi SPECT/CT (index test) compared to histopathology from biopsy or surgical resection (reference test). Interpretation of the index and reference tests will be blinded to the results of the other. Recruitment rate as well as estimates of sensitivity, specificity, positive and negative predictive value will be reported. Semi-structured interviews with patients and clinicians will provide qualitative data to inform onward trial design and delivery. Training materials for ^{99m}Tc-sestamibi SPECT/CT interpretation will be developed, assessed, and optimised. Early health economic modelling using a decision analytic approach for different diagnostic strategies will be performed to understand the potential cost-effectiveness of ^{99m}Tc-sestamibi SPECT/CT.

Ethics and dissemination Ethical approval has been granted (UK HRA REC 20/YH/0279). Study outputs will be presented and published nationally and internationally.

Trial registration ISRCTN12572202; Pre-results

Strengths and Limitations

- MULTI-MIBI is the first multi-centre prospective study to assess ^{99m}Tc-sestamibi SPECT/CT in the evaluation of indeterminate renal tumours
- A composite reference standard of biopsy or surgical pathology allows generalisability of results to patients unwilling or unable to undergo surgical resection
- Blinding of clinicians interpreting index and reference tests reduces risk of bias
- Possible study limitations include the risk of non-diagnostic renal tumour biopsies and tumour misclassification on biopsy.
- If the primary outcome (successful recruitment) is met, this will inform a large-scale multi-centre study

For peer review only

INTRODUCTION

The widespread use of cross-sectional imaging has led to an increase in the incidental detection of renal tumours (1). Based on data from surgical series, it is estimated that up to 30% of renal tumours are benign(2), with an increasing prevalence of benign histology with decreasing tumour size (3). The most common type of benign tumour is the oncocytoma. Unlike renal cell carcinoma (RCC), which commonly requires treatment, renal oncocytomas can be safely managed expectantly (4–6). However, a critical challenge lies in the identification of benign renal tumours, as traditional anatomic imaging techniques such as ultrasound, CT, and MRI are unable to reliably distinguish between the various renal tumour histologies. Although renal mass biopsy can help in this regard, the relatively high non-diagnostic rate (~15%) and associated risk of complications with this procedure has led to its limited adoption in clinical practice (7,8). Thus, the majority of patients presenting with an incidental renal mass undergo treatment for a presumed cancer, exposing those with benign tumours to unnecessary surgical risk while consuming significant health resources (9).

Investigation of new imaging approaches to improve characterisation of incidentally detected small renal masses has been identified as a priority research need by the Renal Cancer Gap Analysis Collaborative, a group composed of clinicians, researchers, patients and caregivers (10). In recent years, ^{99m}Tc-sestamibi SPECT/CT has emerged as a promising non-invasive tool for the identification of benign renal oncocytomas. ^{99m}Tc-sestamibi is a lipophilic cationic radiopharmaceutical that readily accumulates in cells with high concentrations of mitochondria, such as renal oncocytomas (11). Conversely, most histologic subtypes of RCC are relatively devoid of mitochondria and express membrane multi-drug resistance pumps which are known to actively export ^{99m}Tc-sestamibi out of cells (11). These biological differences result in oncocytomas appearing avid, or “hot” and RCCs non-avid, or “cold” on MIBI-kidney studies. A systematic review and meta-analysis including 117 renal lesions from single-centre studies showed pooled sensitivity and specificity of MIBI-kidney to detect renal oncocytomas versus other renal lesions was 92% (95% CI 72-98%) and 88% (95% CI 79-94%), respectively (12). No previous trials of MIBI-kidney have been conducted in the United Kingdom (UK), and there have been no multi-centre trials.

One potential limitation of ^{99m}Tc-sestambi SPECT/CT imaging of renal tumours is that a subset of RCCs exhibit relatively high intracellular concentrations of mitochondria and therefore display uptake of the radiotracer (13–15). These tumours include the chromophobe subtype of RCC, and other oncocytic/chromophobe RCC (16). It is reassuring to note, that these tumours exhibit generally indolent behaviour and low metastatic potential with excellent outcomes on active surveillance (17). We therefore termed this group of tumours as oncocytic renal neoplasms of low malignant potential and suggest that with few exceptions identification of such cT1 tumours on ^{99m}Tc-sestamibi SPECT/CT should be managed similarly to that of benign renal oncocytomas.

Given the excellent performance characteristics of ^{99m}Tc-sestamibi SPECT/CT for the non-invasive identification of renal oncocytomas and oncocytic renal neoplasms of low malignant potential, there is interest in utilizing this test within the UK National Health System (NHS). However, the literature on ^{99m}Tc-sestamibi SPECT/CT remains limited to single centres reporting relatively few tumours. We have recently reported on a pump-

priming pilot study in the UK (18). Herein, we present the protocol for our feasibility study with the following aims: (1) to evaluate the feasibility of a large scale, UK-based, multi-centre, clinical trial of ^{99m}Tc-sestamibi SPECT/CT in the diagnostic pathway for renal tumours and (2) to obtain estimates of sensitivity and specificity with which to power a larger scale trial.

METHODS AND ANALYSIS

Study methods are reported with reference to Standard Protocol Items: Recommendations for Interventional Trials Checklist (SPIRIT) (19) and SPIRIT-Path extension for cellular and molecular pathology content in clinical trial protocols (20).

Study Design

A prospective, multi-centre study to assess the feasibility and diagnostic performance characteristics of ^{99m}TC-sestamibi SPECT/CT in adults (n = 50) with solid, enhancing clinical renal tumours (2-7cm) on cross-sectional imaging. The study design is summarised in Figure 1.

Objectives and outcomes

The primary aim of the study is to evaluate whether a multi-centre diagnostic test evaluation study of ^{99m}Tc-sestamibi SPECT/CT can recruit successfully. Secondary aims are to assess patient and clinician acceptability, refine inclusion/exclusion criteria, sample size requirements and determine clinician training needs for ^{99m}Tc-sestamibi SPECT/CT interpretation.

The study objectives are to determine:

- Will patients consent to have a ^{99m}Tc-sestamibi SPECT/CT prior to surgery or biopsy, including those from under-represented and under-served groups?
- What factors influence patient's decisions to participate?
- What are the perceptions of clinicians and patients of ^{99m}Tc-sestamibi SPECT/CT?
- What barriers and facilitators are there for adoption of ^{99m}Tc-sestamibi SPECT/CT?
- What is the potential cost-effectiveness of using ^{99m}Tc-sestamibi SPECT/CT within the NHS?
- What are the minimally acceptable criteria (MAC) for the sensitivity and specificity of ^{99m}Tc-sestamibi SPECT/CT?
- Is it feasible to train nuclear medicine clinicians across the UK, including those serving under-represented and under-served communities, to interpret ^{99m}Tc-sestamibi SPECT/CT?

The study outcomes are as follows:

Primary outcome

- Recruitment rate

Secondary outcomes

- Sensitivity and specificity of MIBI to detect benign lesions in this study

For peer review only

- Define the MAC for MIBI-kidney to be adopted in clinical practice, to inform the design and parameters of the future definitive clinical trial.
- Interobserver variability and training requirements in the interpretation of MIBI-kidney (local and central reports will be compared)
- Patient and clinician perceptions of utility and experience of MIBI-kidney scans and training
- The evidence requirements for a cost effectiveness analysis

Study Setting
The study will be conducted in 3-6 NHS hospitals in England.

Eligibility Criteria
Consecutive patients discussed at specialist multi-disciplinary team meetings will be screened for eligibility over a planned 15-month recruitment period. The inclusion criteria for entry to the study are adult patients ($\geq$ 18 years) of any gender with a clinical T1 indeterminate solid renal tumour (2-7 cm) on cross-sectional imaging, willing and able to provide informed consent. Patients will be required to have surgery or renal tumour biopsy planned as part of their standard clinical care. Patients entering watchful waiting or active surveillance pathways without histologic diagnosis will be excluded. Other exclusion criteria will include cystic tumours, pregnant and breastfeeding patients, those with a known allergy to ^{99m}Tc-sestamibi and those unwilling or unable to undergo the study procedures.

Test Methods

Index Test
Nuclear medicine clinicians involved in the study will receive study-specific training on the interpretation of ^{99m}TC-sestamibi SPECT/CT from international experts at the beginning of the recruitment period. The training will include a lecture on ^{99m}TC-sestamibi SPECT/CT principles, "hands-on training" supported by experienced faculty and a pre- and post-course assessment.

900 MBq of ^{99m}Tc-sestamibi will be injected intravenously in a single bolus, 75 min before SPECT/CT acquisition of the abdomen with the superior extent of the field-of-view set to the top of the liver dome. CT and SPECT image acquisition will follow manufacturer instructions and local experience. At minimum, we suggest that participating centres have SPECT/CT systems with the following specifications: at least 2-slice helical diagnostic CT scanner, available low-energy all-purpose or low-energy high-resolution collimator, gamma camera or digital detector elements appropriate for 140-kEv photopeak acquisition, and manufacturer-derived iterative reconstruction that includes scatter and attenuation correction.

The reporting clinician will document a qualitative assessment of the tumour as avid, non-avid or indeterminate on reconstructed SPECT/CT images, blinded to clinical information and the result of the histopathology reference test. A spherical region of interest will be drawn to measure maximum uptake in attenuation-corrected images within a) the tumour and b) the ipsilateral renal parenchyma. A ratio of maximum uptake between the tumour and normal renal parenchyma will be calculated. All ^{99m}TC-sestamibi SPECT/CT scans will be

For peer review only

transferred for central review at the lead site (Royal Free Hospital) and discordant reports resolved by discussion and consensus. Local site clinicians will report a subset of studies a second time at the end of the recruitment period to allow assessment of intra-rater reliability.

Reference Test

Histopathology from the final surgical resection specimen is considered the 'gold standard' diagnostic test to determine renal tumour subtype. It is worth noting that although biopsy allows for histological diagnosis, questions remain about the accuracy of this technique for determining the precise histology of a renal tumour, mostly relating to an approximately 15% non-diagnostic rate of this procedure (7,8) and the need for architectural findings in the tissue sample to definitively diagnose some tumour types (21). Despite this, we feel a composite reference standard of surgery and biopsy allows generalisability of the results to non-surgical populations. To maximize the accuracy of this procedure, tumour biopsy will be performed using an image-guided approach by an interventional radiologist experienced in the technique. In the case of a non-diagnostic biopsy the patient will be offered a second attempt, according to local guidelines.

Histopathological reporting of both biopsy and surgical samples will be performed by qualified pathologists at collaborating sites in accordance with the current World Health Organisation classification system for renal tumours (16), as per standard care. Pathologists will be blinded to the ^{99m}Tc-sestamibi SPECT/CT result. Pathology slides/images will be exported for central review by a specialist uro-oncology pathologist and archiving at the lead site (Royal Free Hospital).

Sample size and recruitment

The aim of this study is to assess the feasibility of a multi-centre study of ^{99m}TC-sestamibi SPECT/CT in the diagnostic pathway for renal tumours. Data from the feasibility phase will be used to inform the design and sample size of the definitive trial. We will aim to recruit 50 patients from 3-6 centres. This sample size will allow us to assess if 80% (95% CI 70-90%) of approached patients agree to undergo the study scan. Additionally, this sample size will have sufficient power to detect if there is a significant difference in the estimates of sensitivity between our study population and those reported in the literature. A sample size of 40 patients would achieve 81% power to detect a sensitivity of 0.65 (representing an estimate outside the lower end of the 95% confidence interval for sensitivity from the literature) using a two-sided binomial test at the 5% two-sided alpha level. A 20% inflation to 50 patients, will allow for possible dropouts and other methodological challenges.

Analysis

Qualitative Study of Feasibility and Acceptability

Qualitative data obtained from semi-structured interviews (conducted either by telephone or on virtual platforms e.g. Microsoft Teams) with patients, carers and staff will be combined with documentary analysis (reports, meeting minutes) and will be used to inform within trial decision-making processes via a rapid feedback evaluation approach (22). Transcripts and key documents will be imported into NVivo and analysed using framework analysis (23). Data collection and analysis will be carried out in parallel and emerging

For peer review only - http://bmjopen.bmj.com/site/about/guidelines.xhtml

findings will be shared with the trial team on a monthly basis to inform trial design and delivery.

The findings from the interviews and documentary analysis will be used to develop a discrete choice experiment to gain an understanding of preferences for trial participation and how participants trade-off different attributes of ^{99m}TC-sestamibi SPECT/CT with other management scenarios. In addition, a survey will be conducted – informed by a rapid review of survey instruments reported in the published literature to capture the acceptability of interventions in clinical trials, to provide insights on the barriers or facilitators to patient decision making and determine the degree of acceptability of ^{99m}TC-sestamibi SPECT/CT.

Study of Diagnostic Accuracy

Diagnostic accuracy of ^{99m}Tc-sestamibi SPECT/CT will be estimated by generating 2x2 tables for both avid and non-avid qualitative assessment, and relative radiotracer uptake ratio >0.6 and ≤ 0.6 for external validation of a pre-defined threshold from the literature (24). Analysis of a range of relative uptake ratios will be explored to assess performance at different thresholds. Diagnostic accuracy of ^{99m}TC-sestamibi SPECT/CT will be calculated in terms of sensitivity, specificity and predictive values along with their 95% confidence intervals. The prevalence of renal oncocytoma and other histology subtypes will be calculated with a 95% confidence interval.

Inconclusive test results will be reported(25). The proportion of participants with invalid ^{99m}TC-sestamibi SPECT/CT results e.g. due to technical failure will be reported. The proportion of valid but inconclusive results will also be reported, and their impact on estimates will be assessed by including them as either test positive or test negative in sensitivity analyses. This is to inform how ^{99m}Tc-sestamibi SPECT/CT might be used in the diagnostic pathway. If intended as a replacement test for histopathology, a valid but indeterminate ^{99m}Tc-sestamibi SPECT/CT would be considered non-avid to avoid misclassifying malignant tumours as benign. If however ^{99m}Tc-sestamibi SPECT/CT were to be used as a triage test, where avid tumours undergo confirmatory biopsy, then an indeterminate test could be considered avid to reduce the risk of surgery for benign pathology. The proportion of patients who do not complete the study schedule defined in the protocol will be calculated.

We will assess inter-rater and intra-rater agreement using percentage agreement and Gwet's first-order agreement coefficient(26).

We do not anticipate the need to adjust for diagnostic drift for the reference test, given the short study duration. However, if current pathologic guidelines for renal neoplasia are updated during the course of the study archived samples will be re-reviewed and reported according to the latest guidelines.

Study of Health Economics

For peer review only

Health economic modelling will be used to understand the potential cost-effectiveness of ^{99m}TC-sestamibi SPECT/CT in the evaluation of patients presenting with an indeterminate renal mass (27). A decision analytic approach will compare the following scenarios:

1) Patients have empiric surgery (current standard-of-care).
2) Patients undergo tumour biopsy, those consistent with cancer have surgery and those with benign histology have active surveillance.
3) Patients undergo ^{99m}Tc-sestamibi SPECT/CT, those with a 'cold' scan (suggestive of cancer) have surgery and those with a 'hot' scan (suggestive of benign tumour) have active surveillance.
4) Patients undergo ^{99m}Tc-sestamibi SPECT/CT, those with a 'cold' scan have surgery, and those with a 'hot' scan have a confirmatory biopsy (MIBI would be likened to a triage test to select patients for biopsy for tissue confirmation before embarking on active surveillance).

The model will be populated with evidence from trial and published literature (28). Where data are not available, an expert elicitation approach will be employed to provide parameter values(29). The analysis will then compare the different approaches to standard-of-care by estimating the incremental cost effectiveness ratios (ICERs) and assessing the uncertainty of these estimates using value of information (VOI) analysis. The VOI analysis will quantify the potential value of further research, identify areas of study with the greatest potential benefit and generate recommendations on future study designs.

Data collection

Case report forms (CRFs) in paper and electronic format will be trialled. The CRFs will not bear the participant's name or other directly identifiable data. The participants' study ID will be used for identification purposes. Study-related procedures will be carried out during the baseline routine clinical visit, ^{99m}Tc-sestamibi SPECT/CT visit, and thereafter by telephone or email according to participant preference, as shown in table 1. CCRFs will be checked for completeness and accuracy by designated individuals against source data. Study data captured in paper format will be transcribed to an electronic database. Quality of life data will be captured using the previously validated EQ-5D-5L instrument(30). No analysis will begin until accuracy of the data has been assured.

A participant may withdraw their consent to participate at any time prior to the ^{99m}Tc-sestamibi SPECT/CT scan. The decision to withdraw will be recorded in the CRF and medical notes. Participants withdrawing prior to ^{99m}Tc-sestamibi SPECT/CT will be replaced. If following ^{99m}Tc-sestamibi SPECT/CT the participant states they do not wish to participate in scheduled follow up (EQ-5D-5L completion), or deviate from the protocol, then data already collected will be kept and analysed. These patients will not be replaced.

Baseline data items will include the following:

- Baseline demographics (Age, gender, ethnicity, medical and surgical history, current medication and allergies)
- Baseline blood test results (full blood count, renal function, coagulation screen)
- Baseline imaging (multi-phase [to include non-contrast, arterial-phase, venous-phase, and delayed-phase], contrast-enhanced CT or MRI of the abdomen)

For peer review only

- Renal tumour characteristics (complexity scoring, location, number of lesions)
- Quality of life questionnaire (EQ-5D-5L)

The following data on resource use will be collected at the time of the intervention

- Duration of visit to nuclear medicine department
- Adverse events during and immediately post-MIBI-kidney

The following data will be collected at post-intervention follow-up by telephone or email

- Adverse events following MIBI-kidney
- Quality of life questionnaire (EQ-5D-5L)

After participation in the trial participants will continue follow-up as per standard care.

Patient and Public Involvement

Patient and public involvement has been central to the project concept and design. A pre-study PPI focus group informed the trial protocol and plain English summary. An online PPI survey received 231 responses and indicated 90% would be willing to participate in the proposed study. In addition to the qualitative workstream, PPI representatives from Kidney Cancer UK will form a study support group, meeting at regular intervals throughout the trial to provide advice and input on any trial challenges and developing/approving dissemination materials.

Harms

^{99m}Tc-Sestamibi has been used for cardiac and parathyroid imaging globally for decades and is known to be a safe radiopharmaceutical. The radiation exposure from one MIBI-kidney scan is 14 mSv, equivalent to approximately 5 years of average UK background radiation(31). As MIBI-kidney is the only study intervention in addition to standard care, a data-monitoring committee will not be required.

All adverse events (AE), whether related or unrelated to MIBI-kidney will be documented in the patient's notes, study CRF and the AE log. The AE Log will be sent to the Sponsor at least once per year. Incidental clinically significant abnormalities identified on MIBI-kidney will be recorded as AEs and communicated to the referring clinician and patient. All serious AEs will be recorded on a SAE form and reported to the Sponsor and relevant REC within 15 working days of the chief investigator becoming aware of the event.

Auditing

Investigators and sites will permit trial-related monitoring, audit, REC review and regulatory inspection(s), and provide access to required data and documents.

Ethics and dissemination

Ethical approval for this study has been granted (UK HRA REC 20/YH/0279). Protocol amendments will be promptly disseminated to Sponsor, investigators, and trial steering committee members. The study is recorded on the trial registration website (ISRCTN12572202). The trial involves the administration of unsealed radioactive substances. An Administration of Radioactive Substances Advisory Committee (ARSAC) certificate has been granted (AA-3990).

For peer review only

Study outputs will be presented at national and international conferences and published in peer-reviewed journals. Patient representatives will be involved in output dissemination to the public individual trial participants via study newsletter.

Appendices
Informed consent materials

Table 1: Visit schedule and assessments

Procedures	Screening	Baseline	Intervention	24-72 hour follow-up	Follow-up (standard of care)	Interview follow up
**Demographics**		X				
**Medical history**	X	X				
**Consent**		X				
**Imaging**	X					
**^{99m}Tc-sestamibi SPECT/CT**			X			
**QoL questionnaire**		X		X		
**Adverse event reporting**			X	X		
**Histology test and result**					X	
**Semi-structured interview**						X

Acknowledgements: We are grateful for the invaluable contribution provided by patient representatives.

HW is funded by The Urology Foundation and Pan London Cancer Alliance (Royal Marsden Partners, North Central London Cancer Alliance, North East London Cancer Alliance, South East London Cancer Alliance and the NIHR BRCs). VK receives funding from Prostate Cancer UK and the John Black Charitable Foundation. GDS and IAM are supported by the NIHR Cambridge Biomedical Research Centre (BRC-1215-20014) and GDS by the Cancer Research UK Cambridge Centre [C9685/A25177]. EP is supported by the NIHR Collaboration for Leadership in Applied Health Research and Care (CLAHRC) North Thames at Bart's Health NHS Trust. Mark Emberton receives research support from the United Kingdom's National Institute of Health Research (NIHR) UCLH / UCL Biomedical Research Centre. He was conferred NIHR Senior Investigator Status in 2015. MGBT receives research funding from NIHR, St Peter's Trust, Royal Free Charity, RCS, Facing up 2 Kidney Cancer and Kidney Cancer UK. The views expressed are those of the authors and not necessarily those of the funders.

Authors' contributions: MGBT conceived the study and is the chief investigator and grant holder. HW and MGBT drafted the study protocol. HW, TW, MAG, SPR, BFH, DP, SES, RB, PP, FM, AB, VK, CM, NC, JC, AS, FH, TSOB, GDS, IAM, SD, AA, WW, TMW, CVP, EP, HMD, PL, KG, ME and MGBT have contributed to study design, protocol writing and design of trial documents. SES was responsible for pathology content of the trial protocol. The Sponsor

For peer review only

assisted with protocol writing and design of trial documents. The funding source was not involved in the trial design, protocol writing or design of trial documents and will not participate in its execution, analysis or interpretation. HW and MGBT drafted the manuscript and all other authors contributed to and approved the final document. Publications will be subject to the permission of the Sponsor. Responsibility to report on trial results will lie solely with the authors.

Funding statement: This study is funded by the NIHR Research for Patient Benefit programme (NIHR203545). The views expressed are those of the author(s) and not necessarily those of the NIHR or the Department of Health and Social Care.

Competing interests statement:

GDS has received educational grants from Pfizer, AstraZeneca and Intuitive Surgical; consultancy fees from Pfizer, Merck, EUSA Pharma and CMR Surgical; Travel expenses from Pfizer and Speaker fees from Pfizer. SD provides educational consultancy for GE Healthcare, Bayer, AAA and AVAANT diagnostics.

References

1. Nayan M, Jewett MA, Finelli A. Uncoupling Diagnosis and Treatment of Incidentally ImagedRenal Masses. JAMA Internal Medicine. 2018;178(5):727–8.
2. Kim JH, Li S, Khandwala Y, Chung KJ, Park HK, Chung BI. Association of Prevalence of Benign Pathologic Findings after Partial Nephrectomy with Preoperative Imaging Patterns in the United States from 2007 to 2014. JAMA Surgery. 2019;154(3):225–31.
3. Fernando A, Fowler S, Brien TO. Nephron-sparing surgery across a nation – outcomes from the British Association of Urological Surgeons 2012 national partial nephrectomy audit. BJU International. 2016;117:874–82.
4. Neves J, Varley R, Agnesi S, Withington J, Rodrigues FB, Warren H, et al. Growth and renal function dynamics of renal oncocytomas on active surveillance: a retrospective cohort analysis. BJU International. 2021;128(6):722–7.
5. Liu S, Lee S, Rashid P, Bangash H, Hamid A, Lau J, et al. Active surveillance is suitable for intermediate term follow-up of renal oncocytoma diagnosed by percutaneous core biopsy. BJU International. 2016;118:30–4.
6. Richard PO, Jewett MAS, Bhatt JR, Evans AJ, Timilsina N, Finelli A. Active Surveillance for Renal Neoplasms with Oncocytic Features is Safe. Journal of Urology. 2016;195(3):581–7.
7. Marconi L, Dabestani S, Lam TB, Hofmann F, Stewart F, Norrie J, et al. Systematic Review and Meta-analysis of Diagnostic Accuracy of Percutaneous Renal Tumour Biopsy. European Urology. 2016;69(4):660–73.
8. Patel HD, Johnson MH, Pierorazio PM, Sozio SM, Sharma R, Iyoha E, et al. Diagnostic accuracy and risks of biopsy in the diagnosis of a renal mass suspicious for localized renal cell carcinoma: Systematic review of the literature. Journal of Urology. 2016 May 1;195(5):1340–7.
9. Johnson DC, Vukina J, Smith AB, Meyer AM, Wheeler SB, Kuo TM, et al. Preoperatively Misclassified, Surgically Removed Benign Renal Masses: A Systematic Review of Surgical Series and United States Population Level Burden Estimate. Journal of Urology. 2015;193(1):30–5.

For peer review only

10. Rossi SH, Blick C, Handforth C, Brown JE, Stewart GD. Essential Research Priorities in Renal Cancer: A Modified Delphi Consensus Statement. European Urology Focus. 2019;6(5):991–8.
11. Rowe SP, Gorin MA, Solnes LB, Ball MW, Choudhary A, Pierorazio PM, et al. Correlation of 99m Tc-sestamibi uptake in renal masses with mitochondrial content and multi-drug resistance pump expression. EJNMMI Research. 2017;7(1):80.
12. Wilson MP, Katlariwala P, Murad MH, Abele J, Mcinnes MDF, Low G. Diagnostic accuracy of 99mTc - sestamibi SPECT / CT for detecting renal oncocytomas and other benign renal lesions : a systematic review and meta - analysis. Abdominal Radiology. 2020;45(8):2532-2541.
13. MA Gorin, SP Rowe, AS Baras, LB Solnes, MW Ball, PM Pierorazio, et al. Prospective evaluation of 99mtc-sestamibi SPECT/CT for the diagnosis of renal oncocytomas and hybrid oncocytic/chromophobe tumors. Journal of Urology. 2016;195(4 SUPPL. 1 PG-e1031):e1031.
14. Asi T, Tuncali MC, Murat T, Alkanat NEI, Hazir B, Kosemehmetoglu K, et al. The role of Tc-99m MIBI scintigraphy in clinical T1 renal mass assessment: Does it have a real benefit? Urologic Oncology: Seminars and Original Investigations. 2020;38(12):937.e11-937.e17.
15. Sistani G, Bjazevic J, Kassam Z, Romsa J, Pautler S. The value of 99mTc-sestamibi single-photon emission computed tomography-computed tomography in the evaluation and risk stratification of renal masses. Canadian Journal of Urology. 2020;Epub ahead(Nov 17).
16. Moch H, Amin MB, Berney DM, Compérat EM, Gill AJ, Hartmann A, et al. The 2022 World Health Organization Classification of Tumours of the Urinary System and Male Genital Organs—Part A: Renal, Penile, and Testicular Tumours. European Urology. 2022 Jul
17. Miller BL, Gettle LM, Roo JR van, Ziemlewicz TJ, Best SL, Wells SA, et al. Comparative Analysis of Surgery, Thermal Ablation, and Active Surveillance for Renal Oncocytic Neoplasms. Urology. 2017;112:92–7.
18. Hannah Warren, Anna-Rita Boydell, Abbas Reza, Deborah Pencharz, Beverley F Holman, Soha El-Sheikh, et al. 99mTc-Sestamibi SPECT/CT for indeterminate renal tumours: a pilot diagnostic accuracy study. BJUI. (In Press).
19. AW Chan AW, Tetzlaff J, Gøtzsche P, Altman D, Mann H, Berlin J, et al. SPIRIT 2013 explanation and elaboration: guidance for protocols of clinical trials. BMJ. 2013;346(e7586).
20. Kendall TJ, Robinson M, Brierley DJ, Lim SJ, O'Connor DJ, Shaaban AM, et al. Guidelines for cellular and molecular pathology content in clinical trial protocols: the SPIRIT-Path extension. The Lancet Oncology. 2021;22(10):e435–45. Available from: http://dx.doi.org/10.1016/S1470-2045(21)00344-2
21. Gorin MA, Rowe SP, Allaf ME. Oncocytic Neoplasm on Renal Mass Biopsy: A Diagnostic Conundrum. Oncology (Williston Park). 2016 May;30(5):426–35.
22. Vindrola-Padros C, Chisnall G, Cooper S, Dowrick A, Djellouli N, Symmons SM, et al. Carrying Out Rapid Qualitative Research During a Pandemic: Emerging Lessons From COVID-19. Qualitative Health Research. 2020;30(14):2192–204.
23. Gale NK, Heath G, Cameron E, Rashid S, Redwood S. Using the framework method for the analysis of qualitative data in multi-disciplinary health research. BMC Medical Research Methodology. 2013;13(117).

For peer review only

24. Gorin MA, Rowe SP, Baras AS, Solnes LB, Ball MW, Pierorazio PM, et al. Prospective Evaluation of 99mTc-sestamibi SPECT/CT for the Diagnosis of Renal Oncocytomas and Hybrid Oncocytic/Chromophobe Tumors. European Urology. 2015;69(3):413–6.
25. Shinkins B, Thompson M, Mallett S, Perera R. Diagnostic accuracy studies : how to report and analyse. British Medical Journal. 2013;2778(May):1–11.
26. Gwet K. Handbook of inter-rater reliability. The definitive guide to measuring the extent of agreement amongst raters. 4th Editio. Gaithersburg M 20886 2696, editor. Advances Analytics, LLC; 2014.
27. Love-Koh J. How Useful Are Early Economic Models? Comment on “Problems and Promises of Health Technologies: The Role of Early Health Economic Modelling.” Int J Health Policy Manag. 2020;9(5):215–7.
28. Su ZT, Patel HD, Huang MM, Meyer AR, Pavlovich CP, Pierorazio PM, et al. Cost-effectiveness Analysis of 99mTc-sestamibi SPECT/CT to Guide Management of Small Renal Masses. European Urology Focus. 2021;7(4):827–34.
29. Sperber D, et al. An expert on every street corner? Methods for eliciting distributions in geographically dispersed opinion pools. Value Health. 2013;16(2):434–7.
30. Janssen A, Pickard A, Golicki D, Gudex C, Niewada M, Scalone L, et al. Measurement properties of the EQ-5D-5L compared to the EQ-5D-3L across eight patient groups: a multi-country study. Qual Life Res. 2013;22(7):1717–27.
31. UK Health Security Agency. Ionising radiation: dose comparisons [Internet]. Guidance Document. 2011 [cited 2022 May 24]. Available from: https://www.gov.uk/government/publications/ionising-radiation-dose-comparisons/ionising-radiation-dose-comparisons

Figure 1: Study flow diagram
Table 1: Visit schedule and assessments

For peer review only

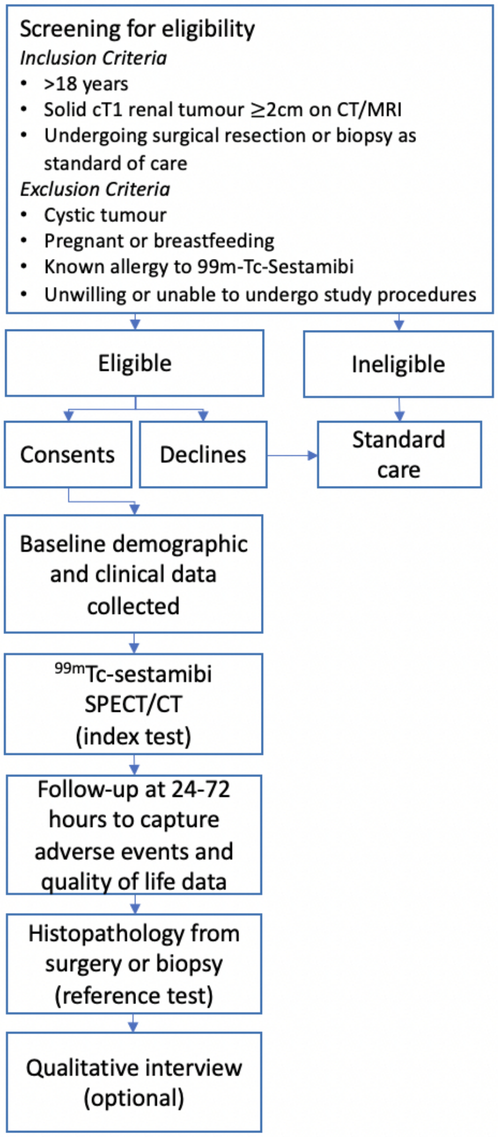

Figure 1: Study flow diagram

92x196mm (144 x 144 DPI)

BMJ Open

Protocol for a MULTI-centre feasibility study to assess the use of 99m Tc-sestaMIBI SPECT/CT in the diagnosis of kidney tumours (MULTI-MIBI study)

Journal:	*BMJ Open*
Manuscript ID	bmjopen-2022-067496.R1
Article Type:	Protocol
Date Submitted by the Author:	06-Oct-2022
Complete List of Authors:	Warren, Hannah; University College London Division of Surgery and Interventional Science,<br>Wagner, Thomas; Royal Free Hospital, Department of Nuclear Medicine<br>Gorin, Michael; Icahn School of Medicine at Mount Sinai, Milton and Carroll Petrie Department of Urology<br>Rowe, Steven; Johns Hopkins University Department of Medicine, Radiology and Radiological Science<br>Holman, Beverley; Royal Free Hospital, Department of Nuclear Medicine<br>Pencharz, Deborah; Royal Free Hospital, Department of Nuclear Medicine<br>El-Sheikh, Soha; Royal Free Hospital, Department of Pathology<br>Barod, Ravi; Royal Free London NHS Foundation Trust, Specialist Centre for Kidney Cancer; University College London, Division of Surgery and Interventional Science<br>Patki, Prasad; Royal Free London NHS Foundation Trust, Specialist Centre for Kidney Cancer<br>Mumtaz, Faiz; Royal Free London NHS Foundation Trust, Specialist Centre for Kidney Cancer<br>Bex, Axel; University College London, Division of Surgery and Interventional Science; Royal Free London NHS Foundation Trust, Specialist Centre for Kidney Cancer<br>Kasivisvanathan, Veeru; University College London, Division Of Surgery and Interventional Science<br>Moore, Caroline; University College London, Division of Surgery and Interventional Science<br>Campain, Nicholas; Royal Devon and Exeter NHS Foundation Trust, Department of Urology<br>Cartledge, Jon; Leeds Teaching Hospitals NHS Trust, Department of Urology<br>Scarsbrook, Andrew; Leeds Teaching Hospitals NHS Trust, Department of Radiology<br>Hassan, Fahim; Guy's and St Thomas' NHS Foundation Trust, Department of Nuclear Medicine<br>O'Brien, Tim; Guy's and St Thomas' NHS Foundation Trust, Department of Urology<br>Stewart, Grant; University of Cambridge, Department of Surgery<br>Mendichovszky, Iosif; University of Cambridge, Department of Radiology; Cambridge University Hospitals NHS Foundation Trust, Department of Nuclear Medicine<br>Dizdarevic, Sabina; University Hospitals Sussex NHS Foundation Trust,

	Department of Nuclear Medicine; Brighton and Sussex Medical School<br>Alanbuki, Ammar; University Hospitals Sussex NHS Foundation Trust, Department of Urology<br>Wildgoose, William; Patient Representative<br>Wah, Tze; Leeds Teaching Hospitals NHS Trust, Department of Radiology<br>Vindrola-Padros, Cecilia; University College London, Rapid Research, Evaluation and Appraisal Lab (RREAL), Department of Targeted Intervention<br>Pizzo, Elena; University College London, Department of Applied Health Research<br>Dehbi, Hakim-Moulay; University College London Institute of Clinical Trials and Methodology<br>Lorgelly, Paula; University College London, Department of Applied Health Research<br>Gurusamy, Kurinchi; University College London, Department of Surgery and Interventional Science<br>Emberton, Mark; University College London, Division of Surgery and Interventional Science; University College Hospital, Department of Urology<br>Tran, Maxine ; University College London, Division of Surgery and Interventional Science; Royal Free Hospital, Specialist Centre for Kidney Cancer
**Primary Subject Heading**:	Urology
Secondary Subject Heading:	Radiology and imaging
Keywords:	Urological tumours < UROLOGY, Nuclear radiology < RADIOLOGY & IMAGING, HEALTH ECONOMICS

SCHOLARONE™
Manuscripts

TITLE PAGE

Title: Protocol for a MULTI-centre feasibility study to assess the use of ^{99m} Tc-sestaMIBI SPECT/CT in the diagnosis of kidney tumours (MULTI-MIBI study)

H Warren[1,2], T Wagner[3], MA Gorin[4], SP Rowe[5], BF Holman[3], D Pencharz[3], S El-Sheikh[2], R Barod[1,2], P Patki[2], F Mumtaz[2], A Bex[2], V Kasivisvanathan[1], C Moore[1], N Campain[6], J Cartledge[7], A Scarsbrook[8], F Hassan[9], TS O'Brien[10], GD Stewart[11], IA Mendichovszky[12,13], S Dizdarevic[14,15], A Alanbuki[16], W Wildgoose[17], TM Wah[8], C Vindrola-Padros[18], E Pizzo[19], HM Dehbi[20], P Lorgelly[18]9 K Gurusamy[1], M Emberton[1] and MGB Tran[1,2]

Corresponding author:
Hannah Warren[1,2]
[1]Division of Surgery and Interventional Science, University College London, UK
[2]Specialist Centre for Kidney Cancer, Royal Free Hospital, London, UK
h.warren@ucl.ac.uk

Affiliations:
[1]Division of Surgery and Interventional Science, University College London, UK
[2]Specialist Centre for Kidney Cancer, Royal Free Hospital, London, UK
[3]Department of Nuclear Medicine, Royal Free Hospital, London, UK
[4] Milton and Carroll Petrie Department of Urology, Icahn School of Medicine at Mount Sinai, New York, NY, USA
[5]Division of Nuclear Medicine and Molecular Imaging, The Russell H. Morgan Department of Radiology and Radiological Sciences, Johns Hopkins University School of Medicine, Baltimore, MD, USA
[6]Department of Urology Royal Devon & Exeter NHS Foundation Trust, UK
[7]Department of Urology, Leeds Teaching Hospitals NHS Trust, Leeds, UK
[8]Department of Radiology, Leeds Teaching Hospitals NHS Trust, Leeds, UK
[9]Department of Nuclear Medicine, Guy's & St Thomas' NHS Foundation Trust, London, UK
[10]Department of Urology, Guy's & St Thomas' NHS Foundation Trust, London, UK
[11]Department of Surgery, University of Cambridge, Cambridge Biomedical Campus, Cambridge, UK
[12]Department of Nuclear Medicine, Cambridge University Hospitals NHS Foundation Trust
[13]Department of Radiology, University of Cambridge
[14]Department of Nuclear Medicine, University Hospitals Sussex NHS Foundation Trust, UK
[15]Brighton and Sussex Medical School, UK
[16]Department of Urology, University Hospitals Sussex NHS Foundation Trust, UK
[17]Patient Advisor
[18]Rapid Research, Evaluation and Appraisal Lab (RREAL), Department of Targeted Intervention, University College London, UK
[19]Department of Applied Health Research, University College London, London, UK
[20]Comprehensive Clinical Trials Unit, Institute of Clinical Trials and Methodology, UCL, London, UK

For peer review only

ABSTRACT

Introduction

The incidence of renal tumours is increasing and anatomic imaging cannot reliably distinguish benign tumours from renal cell carcinoma (RCC). Up to 30% of renal tumours are benign, with oncocytomas the most common type. Biopsy has not been routinely adopted in many centres due to concerns surrounding non-diagnostic rate, bleeding, and tumour seeding. As a result, benign masses are often unnecessarily surgically resected. ^{99m}Tc-sestamibi SPECT/CT has shown high diagnostic accuracy for benign renal oncocytomas and other oncocytic renal neoplasms of low malignant potential in single-centre studies. The primary aim of MULTI-MIBI is to assess feasibility of a multi-centre study of ^{99m}Tc-sestamibi SPECT/CT against a reference standard of histopathology from surgical resection or biopsy. Secondary aims of the study include obtaining estimates of ^{99m}Tc-sestamibi SPECT/CT sensitivity and specificity and to inform the design and conduct of a future definitive trial.

Methods and analysis

A feasibility prospective multi-centre study of participants with indeterminate, clinical T1 renal tumours to undergo ^{99m}Tc-sestamibi SPECT/CT (index test) compared to histopathology from biopsy or surgical resection (reference test). Interpretation of the index and reference tests will be blinded to the results of the other. Recruitment rate as well as estimates of sensitivity, specificity, positive and negative predictive value will be reported. Semi-structured interviews with patients and clinicians will provide qualitative data to inform onward trial design and delivery. Training materials for ^{99m}Tc-sestamibi SPECT/CT interpretation will be developed, assessed, and optimised. Early health economic modelling using a decision analytic approach for different diagnostic strategies will be performed to understand the potential cost-effectiveness of ^{99m}Tc-sestamibi SPECT/CT.

Ethics and dissemination Ethical approval has been granted (UK HRA REC 20/YH/0279) protocol v5.0 dated 21/06/2022. Study outputs will be presented and published nationally and internationally.

Trial registration ISRCTN12572202; Pre-results

Strengths and Limitations

- MULTI-MIBI is the first multi-centre prospective study to assess ^{99m}Tc-sestamibi SPECT/CT in the evaluation of indeterminate renal tumours
- A composite reference standard of biopsy or surgical pathology allows generalisability of results to patients unwilling or unable to undergo surgical resection
- Blinding of clinicians interpreting index and reference tests reduces risk of bias
- Possible study limitations include the risk of non-diagnostic renal tumour biopsies and tumour misclassification on biopsy.
- If the primary outcome (successful recruitment) is met, this will inform a large-scale multi-centre study

For peer review only

INTRODUCTION

The widespread use of cross-sectional imaging has led to an increase in the incidental detection of renal tumours (1). Based on data from surgical series, it is estimated that up to 30% of renal tumours are benign(2), with an increasing prevalence of benign histology with decreasing tumour size (3). The most common type of benign tumour is the oncocytoma. Unlike renal cell carcinoma (RCC), which commonly requires treatment, renal oncocytomas can be safely managed expectantly (4–6). However, a critical challenge lies in the identification of benign renal tumours, as traditional anatomic imaging techniques such as ultrasound, CT, and MRI are unable to reliably distinguish between the various renal tumour histologies. Although renal mass biopsy can help in this regard, the relatively high non-diagnostic rate (~15%) and associated risk of complications with this procedure has led to its limited adoption in clinical practice (7,8). Thus, the majority of patients presenting with an incidental renal mass undergo treatment for a presumed cancer, exposing those with benign tumours to unnecessary surgical risk while consuming significant health resources (9).

Investigation of new imaging approaches to improve characterisation of incidentally detected small renal masses has been identified as a priority research need by the Renal Cancer Gap Analysis Collaborative, a group composed of clinicians, researchers, patients and caregivers (10). In recent years, ^{99m}Tc-sestamibi SPECT/CT has emerged as a promising non-invasive tool for the identification of benign renal oncocytomas. ^{99m}Tc-sestamibi is a lipophilic cationic radiopharmaceutical that readily accumulates in cells with high concentrations of mitochondria, such as renal oncocytomas (11). Conversely, most histologic subtypes of RCC are relatively devoid of mitochondria and express membrane multi-drug resistance pumps which are known to actively export ^{99m}Tc-sestamibi out of cells (11). These biological differences result in oncocytomas appearing avid, or "hot" and RCCs non-avid, or "cold" on MIBI-kidney studies. A systematic review and meta-analysis including 117 renal lesions from single-centre studies showed pooled sensitivity and specificity of MIBI-kidney to detect renal oncocytomas versus other renal lesions was 92% (95% CI 72-98%) and 88% (95% CI 79-94%), respectively (12). No previous trials of MIBI-kidney have been conducted in the United Kingdom (UK), and there have been no multi-centre trials.

One potential limitation of ^{99m}Tc-sestambi SPECT/CT imaging of renal tumours is that a subset of RCCs exhibit relatively high intracellular concentrations of mitochondria and therefore display uptake of the radiotracer (13–15). These tumours include the chromophobe subtype of RCC, and other oncocytic/chromophobe RCC (16). It is reassuring to note, that these tumours exhibit generally indolent behaviour and low metastatic potential with excellent outcomes on active surveillance (17). We therefore termed this group of tumours as oncocytic renal neoplasms of low malignant potential and suggest that with few exceptions identification of such cT1 tumours on ^{99m}Tc-sestamibi SPECT/CT should be managed similarly to that of benign renal oncocytomas.

Given the excellent performance characteristics of ^{99m}Tc-sestamibi SPECT/CT for the non-invasive identification of renal oncocytomas and oncocytic renal neoplasms of low malignant potential, there is interest in utilizing this test within the UK National Health System (NHS). However, the literature on ^{99m}Tc-sestamibi SPECT/CT remains limited to single centres reporting relatively few tumours. We have recently reported on a pump-

For peer review only - http://bmjopen.bmj.com/site/about/guidelines.xhtml

priming pilot study in the UK (18). Herein, we present the protocol for our feasibility study with the following aims: (1) to evaluate the feasibility of a large scale, UK-based, multi-centre, clinical trial of ^{99m}Tc-sestamibi SPECT/CT in the diagnostic pathway for renal tumours and (2) to obtain estimates of sensitivity and specificity with which to power a larger scale trial.

METHODS AND ANALYSIS

Study methods are reported with reference to Standard Protocol Items: Recommendations for Interventional Trials Checklist (SPIRIT) (19) and SPIRIT-Path extension for cellular and molecular pathology content in clinical trial protocols (20).

Study Design

A prospective, multi-centre study to assess the feasibility and diagnostic performance characteristics of ^{99m}TC-sestamibi SPECT/CT in adults (n = 50) with solid, enhancing clinical renal tumours (2-7cm) on cross-sectional imaging. The study design is summarised in Figure 1.

Objectives and outcomes

The primary aim of the study is to evaluate whether a multi-centre diagnostic test evaluation study of ^{99m}Tc-sestamibi SPECT/CT can recruit successfully. Secondary aims are to assess patient and clinician acceptability, refine inclusion/exclusion criteria, sample size requirements and determine clinician training needs for ^{99m}Tc-sestamibi SPECT/CT interpretation.

The study objectives are to determine:

- Will patients consent to have a ^{99m}Tc-sestamibi SPECT/CT prior to surgery or biopsy, including those from under-represented and under-served groups?
- What factors influence patient's decisions to participate?
- What are the perceptions of clinicians and patients of ^{99m}Tc-sestamibi SPECT/CT?
- What barriers and facilitators are there for adoption of ^{99m}Tc-sestamibi SPECT/CT?
- What is the potential cost-effectiveness of using ^{99m}Tc-sestamibi SPECT/CT within the NHS?
- What are the minimally acceptable criteria (MAC) for the sensitivity and specificity of ^{99m}Tc-sestamibi SPECT/CT?
- Is it feasible to train nuclear medicine clinicians across the UK, including those serving under-represented and under-served communities, to interpret ^{99m}Tc-sestamibi SPECT/CT?

The study outcomes are as follows:

Primary outcome

- Recruitment rate

Secondary outcomes

- Sensitivity and specificity of MIBI to detect benign lesions in this study

For peer review only

- Define the MAC for MIBI-kidney to be adopted in clinical practice, to inform the design and parameters of the future definitive clinical trial.
- Interobserver variability and training requirements in the interpretation of MIBI-kidney (local and central reports will be compared)
- Patient and clinician perceptions of utility and experience of MIBI-kidney scans and training
- The evidence requirements for a cost effectiveness analysis

Study Setting

The study will be conducted in 3-6 NHS hospitals in England.

Eligibility Criteria

Consecutive patients discussed at specialist multi-disciplinary team meetings will be screened for eligibility over a planned 15-month recruitment period. The inclusion criteria for entry to the study are adult patients ($\geq$ 18 years) of any gender with a clinical T1 indeterminate solid renal tumour (2-7 cm) on cross-sectional imaging, willing and able to provide informed consent. Patients will be required to have surgery or renal tumour biopsy planned as part of their standard clinical care. Patients entering watchful waiting or active surveillance pathways without histologic diagnosis will be excluded. Other exclusion criteria will include cystic tumours, pregnant and breastfeeding patients, those with a known allergy to ^{99m}Tc-sestamibi and those unwilling or unable to undergo the study procedures.

Test Methods

Index Test

Nuclear medicine clinicians involved in the study will receive study-specific training on the interpretation of ^{99m}Tc-sestamibi SPECT/CT from international experts at the beginning of the recruitment period. The training will include a lecture on ^{99m}Tc-sestamibi SPECT/CT principles, "hands-on training" supported by experienced faculty and a pre- and post-course assessment.

900 MBq of ^{99m}Tc-sestamibi will be injected intravenously in a single bolus, 75 min before SPECT/CT acquisition of the abdomen with the superior extent of the field-of-view set to the top of the liver dome. CT and SPECT image acquisition will follow manufacturer instructions and local experience. At minimum, we suggest that participating centres have SPECT/CT systems with the following specifications: at least 2-slice helical diagnostic CT scanner, available low-energy all-purpose or low-energy high-resolution collimator, gamma camera or digital detector elements appropriate for 140-kEv photopeak acquisition, and manufacturer-derived iterative reconstruction that includes scatter and attenuation correction.

The reporting clinician will document a qualitative assessment of the tumour as avid, non-avid or indeterminate on reconstructed SPECT/CT images, blinded to clinical information and the result of the histopathology reference test. A spherical region of interest will be drawn to measure maximum uptake in attenuation-corrected images within a) the tumour and b) the ipsilateral renal parenchyma. A ratio of maximum uptake between the tumour and normal renal parenchyma will be calculated. All ^{99m}TC-sestamibi SPECT/CT scans will be

For peer review only

transferred for central review at the lead site (Royal Free Hospital) and discordant reports resolved by discussion and consensus. Local site clinicians will report a subset of studies a second time at the end of the recruitment period to allow assessment of intra-rater reliability.

Reference Test

Histopathology from the final surgical resection specimen is considered the 'gold standard' diagnostic test to determine renal tumour subtype. It is worth noting that although biopsy allows for histological diagnosis, questions remain about the accuracy of this technique for determining the precise histology of a renal tumour, mostly relating to an approximately 15% non-diagnostic rate of this procedure (7,8) and the need for architectural findings in the tissue sample to definitively diagnose some tumour types (21). Despite this, we feel a composite reference standard of surgery and biopsy allows generalisability of the results to non-surgical populations. To maximize the accuracy of this procedure, tumour biopsy will be performed using an image-guided approach by an interventional radiologist experienced in the technique. In the case of a non-diagnostic biopsy the patient will be offered a second attempt, according to local guidelines.

Histopathological reporting of both biopsy and surgical samples will be performed by qualified pathologists at collaborating sites in accordance with the current World Health Organisation classification system for renal tumours (16), as per standard care. Pathologists will be blinded to the ^{99m}Tc-sestamibi SPECT/CT result. Pathology slides/images will be exported for central review by a specialist uro-oncology pathologist and archiving at the lead site (Royal Free Hospital).

Sample size and recruitment

The aim of this study is to assess the feasibility of a multi-centre study of ^{99m}TC-sestamibi SPECT/CT in the diagnostic pathway for renal tumours. Data from the feasibility phase will be used to inform the design and sample size of the definitive trial. We will aim to recruit 50 patients from 3-6 centres. This sample size will allow us to assess if 80% (95% CI 70-90%) of approached patients agree to undergo the study scan. Additionally, this sample size will have sufficient power to detect if there is a significant difference in the estimates of sensitivity between our study population and those reported in the literature. A sample size of 40 patients would achieve 81% power to detect a sensitivity of 0.65 (representing an estimate outside the lower end of the 95% confidence interval for sensitivity from the literature) using a two-sided binomial test at the 5% two-sided alpha level. A 20% inflation to 50 patients, will allow for possible dropouts and other methodological challenges.

Analysis

Qualitative Study of Feasibility and Acceptability

Qualitative data obtained from semi-structured interviews (conducted either by telephone or on virtual platforms e.g. Microsoft Teams) with patients, carers and staff will be combined with documentary analysis (reports, meeting minutes) and will be used to inform within trial decision-making processes via a rapid feedback evaluation approach (22). Transcripts and key documents will be imported into NVivo and analysed using framework analysis (23). Data collection and analysis will be carried out in parallel and emerging

For peer review only - http://bmjopen.bmj.com/site/about/guidelines.xhtml

findings will be shared with the trial team on a monthly basis to inform trial design and delivery.

The findings from the interviews and documentary analysis will be used to develop a discrete choice experiment to gain an understanding of preferences for trial participation and how participants trade-off different attributes of ^{99m}TC-sestamibi SPECT/CT with other management scenarios. In addition, a survey will be conducted – informed by a rapid review of survey instruments reported in the published literature to capture the acceptability of interventions in clinical trials, to provide insights on the barriers or facilitators to patient decision making and determine the degree of acceptability of ^{99m}TC-sestamibi SPECT/CT.

Study of Diagnostic Accuracy

Diagnostic accuracy of ^{99m}Tc-sestamibi SPECT/CT will be estimated by generating 2x2 tables for both avid and non-avid qualitative assessment, and relative radiotracer uptake ratio >0.6 and ≤ 0.6 for external validation of a pre-defined threshold from the literature (24). Analysis of a range of relative uptake ratios will be explored to assess performance at different thresholds. Diagnostic accuracy of ^{99m}TC-sestamibi SPECT/CT will be calculated in terms of sensitivity, specificity and predictive values along with their 95% confidence intervals. The prevalence of renal oncocytoma and other histology subtypes will be calculated with a 95% confidence interval.

Inconclusive test results will be reported(25). The proportion of participants with invalid ^{99m}TC-sestamibi SPECT/CT results e.g. due to technical failure will be reported. The proportion of valid but inconclusive results will also be reported, and their impact on estimates will be assessed by including them as either test positive or test negative in sensitivity analyses. This is to inform how ^{99m}Tc-sestamibi SPECT/CT might be used in the diagnostic pathway. If intended as a replacement test for histopathology, a valid but indeterminate ^{99m}Tc-sestamibi SPECT/CT would be considered non-avid to avoid misclassifying malignant tumours as benign. If however ^{99m}Tc-sestamibi SPECT/CT were to be used as a triage test, where avid tumours undergo confirmatory biopsy, then an indeterminate test could be considered avid to reduce the risk of surgery for benign pathology. The proportion of patients who do not complete the study schedule defined in the protocol will be calculated.

We will assess inter-rater and intra-rater agreement using percentage agreement and Gwet's first-order agreement coefficient(26).

We do not anticipate the need to adjust for diagnostic drift for the reference test, given the short study duration. However, if current pathologic guidelines for renal neoplasia are updated during the course of the study archived samples will be re-reviewed and reported according to the latest guidelines.

Study of Health Economics

For peer review only

Health economic modelling will be used to understand the potential cost-effectiveness of ^{99m}TC-sestamibi SPECT/CT in the evaluation of patients presenting with an indeterminate renal mass (27). A decision analytic approach will compare the following scenarios:

1) Patients have empiric surgery (current standard-of-care).
2) Patients undergo tumour biopsy, those consistent with cancer have surgery and those with benign histology have active surveillance.
3) Patients undergo ^{99m}Tc-sestamibi SPECT/CT, those with a 'cold' scan (suggestive of cancer) have surgery and those with a 'hot' scan (suggestive of benign tumour) have active surveillance.
4) Patients undergo ^{99m}Tc-sestamibi SPECT/CT, those with a 'cold' scan have surgery, and those with a 'hot' scan have a confirmatory biopsy (MIBI would be likened to a triage test to select patients for biopsy for tissue confirmation before embarking on active surveillance).

The model will be populated with evidence from trial and published literature (28). Where data are not available, an expert elicitation approach will be employed to provide parameter values(29). The analysis will then compare the different approaches to standard-of-care by estimating the incremental cost effectiveness ratios (ICERs) and assessing the uncertainty of these estimates using value of information (VOI) analysis. The VOI analysis will quantify the potential value of further research, identify areas of study with the greatest potential benefit and generate recommendations on future study designs.

Data collection

Case report forms (CRFs) in paper and electronic format will be trialled. The CRFs will not bear the participant's name or other directly identifiable data. The participants' study ID will be used for identification purposes. Study-related procedures will be carried out during the baseline routine clinical visit, ^{99m}Tc-sestamibi SPECT/CT visit, and thereafter by telephone or email according to participant preference, as shown in table 1. CCRFs will be checked for completeness and accuracy by designated individuals against source data. Study data captured in paper format will be transcribed to an electronic database. Quality of life data will be captured using the previously validated EQ-5D-5L instrument(30). No analysis will begin until accuracy of the data has been assured. The final trial dataset will be accessible to the chief investigator, statistician and health economist.

A participant may withdraw their consent to participate at any time prior to the ^{99m}Tc-sestamibi SPECT/CT scan. The decision to withdraw will be recorded in the CRF and medical notes. Participants withdrawing prior to ^{99m}Tc-sestamibi SPECT/CT will be replaced. If following ^{99m}Tc-sestamibi SPECT/CT the participant states they do not wish to participate in scheduled follow up (EQ-5D-5L completion), or deviate from the protocol, then data already collected will be kept and analysed. These patients will not be replaced.

Baseline data items will include the following:

- Baseline demographics (Age, gender, ethnicity, medical and surgical history, current medication and allergies)
- Baseline blood test results (full blood count, renal function, coagulation screen)

For peer review only - http://bmjopen.bmj.com/site/about/guidelines.xhtml

- Baseline imaging (multi-phase [to include non-contrast, arterial-phase, venous-phase, and delayed-phase], contrast-enhanced CT or MRI of the abdomen)
- Renal tumour characteristics (complexity scoring, location, number of lesions)
- Quality of life questionnaire (EQ-5D-5L)

The following data on resource use will be collected at the time of the intervention

- Duration of visit to nuclear medicine department
- Adverse events during and immediately post-MIBI-kidney

The following data will be collected at post-intervention follow-up by telephone or email

- Adverse events following MIBI-kidney
- Quality of life questionnaire (EQ-5D-5L)

After participation in the trial participants will continue follow-up as per standard care.

Patient and Public Involvement
Patient and public involvement has been central to the project concept and design. A pre-study PPI focus group informed the trial protocol and plain English summary. An online PPI survey received 231 responses and indicated 90% would be willing to participate in the proposed study. In addition to the qualitative workstream, PPI representatives from Kidney Cancer UK will form a study support group, meeting at regular intervals throughout the trial to provide advice and input on any trial challenges and developing/approving dissemination materials.

Harms
^{99m}Tc-Sestamibi has been used for cardiac and parathyroid imaging globally for decades and is known to be a safe radiopharmaceutical. The radiation exposure from one MIBI-kidney scan is 14 mSv, equivalent to approximately 5 years of average UK background radiation(31). As MIBI-kidney is the only study intervention in addition to standard care, a data-monitoring committee will not be required.

All adverse events (AE), whether related or unrelated to MIBI-kidney will be documented in the patient's notes, study CRF and the AE log. The AE Log will be sent to the Sponsor (University College London & University College London Hospitals Joint Research Office) at least once per year. Incidental clinically significant abnormalities identified on MIBI-kidney will be recorded as AEs and communicated to the referring clinician and patient. All serious AEs will be recorded on a SAE form and reported to the Sponsor and relevant REC within 15 working days of the chief investigator becoming aware of the event.

Auditing
Investigators and sites will permit trial-related monitoring, audit, REC review and regulatory inspection(s), and provide access to required data and documents.

Ethics and dissemination
Ethical approval for this study has been granted (UK HRA REC 20/YH/0279). Protocol amendments will be promptly disseminated to Sponsor, investigators, and trial steering committee members. The study is recorded on the trial registration website (ISRCTN12572202). The trial involves the administration of unsealed radioactive substances. An Administration of Radioactive Substances Advisory Committee (ARSAC) certificate has been granted (AA-3990).

For peer review only

Study outputs will be presented at national and international conferences and published in peer-reviewed journals. Patient representatives will be involved in output dissemination to the public individual trial participants via study newsletter.

Appendices
Informed consent materials

Table 1: Visit schedule and assessments

Procedures	Screening	Baseline	Intervention	24-72 hour follow-up	Follow-up (standard of care)	Interview follow up
**Demographics**		X				
**Medical history**	X	X				
**Consent** (obtained by clinician/research nurse)		X				
**Imaging**	X					
**^{99m}Tc-sestamibi SPECT/CT**			X			
**QoL questionnaire**		X		X		
**Adverse event reporting**			X	X		
**Histology test and result**					X	
**Semi-structured interview**						X

Acknowledgements: We are grateful for the invaluable contribution provided by patient representatives.

HW is funded by The Urology Foundation and Pan London Cancer Alliance (Royal Marsden Partners, North Central London Cancer Alliance, North East London Cancer Alliance, South East London Cancer Alliance and the NIHR BRCs). VK receives funding from Prostate Cancer UK and the John Black Charitable Foundation. GDS and IAM are supported by the NIHR Cambridge Biomedical Research Centre (BRC-1215-20014) and GDS by the Cancer Research UK Cambridge Centre [C9685/A25177]. EP is supported by the NIHR Collaboration for Leadership in Applied Health Research and Care (CLAHRC) North Thames at Bart's Health NHS Trust. Mark Emberton receives research support from the United Kingdom's National Institute of Health Research (NIHR) UCLH / UCL Biomedical Research Centre. He was conferred NIHR Senior Investigator Status in 2015. MGBT receives research funding from NIHR, St Peter's Trust, Royal Free Charity, RCS, Facing up 2 Kidney Cancer and Kidney Cancer UK. The views expressed are those of the authors and not necessarily those of the funders.

For peer review only - http://bmjopen.bmj.com/site/about/guidelines.xhtml

Authors' contributions: MGBT conceived the study and is the chief investigator and grant holder. HW and MGBT drafted the study protocol. HW, TW, MAG, SPR, BFH, DP, SES, RB, PP, FM, AB, VK, CM, NC, JC, AS, FH, TSOB, GDS, IAM, SD, AA, WW, TMW, CVP, EP, HMD, PL, KG, ME and MGBT have contributed to study design, protocol writing and design of trial documents. SES was responsible for pathology content of the trial protocol. The Sponsor assisted with protocol writing and design of trial documents. The funding source was not involved in the trial design, protocol writing or design of trial documents and will not participate in its execution, analysis or interpretation. HW and MGBT drafted the manuscript and all other authors contributed to and approved the final document. Publications will be subject to the permission of the Sponsor. Responsibility to report on trial results will lie solely with the authors.

Funding statement: This study is funded by the NIHR Research for Patient Benefit programme (NIHR203545). The views expressed are those of the author(s) and not necessarily those of the NIHR or the Department of Health and Social Care.

Competing interests statement:
GDS has received educational grants from Pfizer, AstraZeneca and Intuitive Surgical; consultancy fees from Pfizer, Merck, EUSA Pharma and CMR Surgical; Travel expenses from Pfizer and Speaker fees from Pfizer. SD provides educational consultancy for GE Healthcare, Bayer, AAA and AVAANT diagnostics.

References

1. Nayan M, Jewett MA, Finelli A. Uncoupling Diagnosis and Treatment of Incidentally ImagedRenal Masses. JAMA Internal Medicine. 2018;178(5):727–8.
2. Kim JH, Li S, Khandwala Y, Chung KJ, Park HK, Chung BI. Association of Prevalence of Benign Pathologic Findings after Partial Nephrectomy with Preoperative Imaging Patterns in the United States from 2007 to 2014. JAMA Surgery. 2019;154(3):225–31.
3. Fernando A, Fowler S, Brien TO. Nephron-sparing surgery across a nation – outcomes from the British Association of Urological Surgeons 2012 national partial nephrectomy audit. BJU International. 2016;117:874–82.
4. Neves J, Varley R, Agnesi S, Withington J, Rodrigues FB, Warren H, et al. Growth and renal function dynamics of renal oncocytomas on active surveillance: a retrospective cohort analysis. BJU International. 2021;128(6):722–7.
5. Liu S, Lee S, Rashid P, Bangash H, Hamid A, Lau J, et al. Active surveillance is suitable for intermediate term follow-up of renal oncocytoma diagnosed by percutaneous core biopsy. BJU International. 2016;118:30–4.
6. Richard PO, Jewett MAS, Bhatt JR, Evans AJ, Timilsina N, Finelli A. Active Surveillance for Renal Neoplasms with Oncocytic Features is Safe. Journal of Urology. 2016;195(3):581–7.
7. Marconi L, Dabestani S, Lam TB, Hofmann F, Stewart F, Norrie J, et al. Systematic Review and Meta-analysis of Diagnostic Accuracy of Percutaneous Renal Tumour Biopsy. European Urology. 2016;69(4):660–73.
8. Patel HD, Johnson MH, Pierorazio PM, Sozio SM, Sharma R, Iyoha E, et al. Diagnostic accuracy and risks of biopsy in the diagnosis of a renal mass suspicious for localized renal cell carcinoma: Systematic review of the literature. Journal of Urology. 2016 May 1;195(5):1340–7.

For peer review only - http://bmjopen.bmj.com/site/about/guidelines.xhtml

9. Johnson DC, Vukina J, Smith AB, Meyer AM, Wheeler SB, Kuo TM, et al. Preoperatively Misclassified, Surgically Removed Benign Renal Masses: A Systematic Review of Surgical Series and United States Population Level Burden Estimate. Journal of Urology. 2015;193(1):30–5.
10. Rossi SH, Blick C, Handforth C, Brown JE, Stewart GD. Essential Research Priorities in Renal Cancer: A Modified Delphi Consensus Statement. European Urology Focus. 2019;6(5):991–8.
11. Rowe SP, Gorin MA, Solnes LB, Ball MW, Choudhary A, Pierorazio PM, et al. Correlation of 99m Tc-sestamibi uptake in renal masses with mitochondrial content and multi-drug resistance pump expression. EJNMMI Research. 2017;7(1):80.
12. Wilson MP, Katlariwala P, Murad MH, Abele J, Mcinnes MDF, Low G. Diagnostic accuracy of 99mTc - sestamibi SPECT / CT for detecting renal oncocytomas and other benign renal lesions : a systematic review and meta - analysis. Abdominal Radiology. 2020;45(8):2532-2541.
13. MA Gorin, SP Rowe, AS Baras, LB Solnes, MW Ball, PM Pierorazio, et al. Prospective evaluation of 99mtc-sestamibi SPECT/CT for the diagnosis of renal oncocytomas and hybrid oncocytic/chromophobe tumors. Journal of Urology. 2016;195(4 SUPPL. 1 PG-e1031):e1031.
14. Asi T, Tuncali MC, Murat T, Alkanat NEI, Hazir B, Kosemehmetoglu K, et al. The role of Tc-99m MIBI scintigraphy in clinical T1 renal mass assessment: Does it have a real benefit? Urologic Oncology: Seminars and Original Investigations. 2020;38(12):937.e11-937.e17.
15. Sistani G, Bjazevic J, Kassam Z, Romsa J, Pautler S. The value of 99mTc-sestamibi single-photon emission computed tomography-computed tomography in the evaluation and risk stratification of renal masses. Canadian Journal of Urology. 2020;Epub ahead(Nov 17).
16. Moch H, Amin MB, Berney DM, Compérat EM, Gill AJ, Hartmann A, et al. The 2022 World Health Organization Classification of Tumours of the Urinary System and Male Genital Organs—Part A: Renal, Penile, and Testicular Tumours. European Urology. 2022 Jul
17. Miller BL, Gettle LM, Roo JR van, Ziemlewicz TJ, Best SL, Wells SA, et al. Comparative Analysis of Surgery, Thermal Ablation, and Active Surveillance for Renal Oncocytic Neoplasms. Urology. 2017;112:92–7.
18. Hannah Warren, Anna-Rita Boydell, Abbas Reza, Deborah Pencharz, Beverley F Holman, Soha El-Sheikh, et al. 99mTc-Sestamibi SPECT/CT for indeterminate renal tumours: a pilot diagnostic accuracy study. BJUI. (In Press).
19. AW Chan AW, Tetzlaff J, Gøtzsche P, Altman D, Mann H, Berlin J, et al. SPIRIT 2013 explanation and elaboration: guidance for protocols of clinical trials. BMJ. 2013;346(e7586).
20. Kendall TJ, Robinson M, Brierley DJ, Lim SJ, O'Connor DJ, Shaaban AM, et al. Guidelines for cellular and molecular pathology content in clinical trial protocols: the SPIRIT-Path extension. The Lancet Oncology. 2021;22(10):e435–45. Available from: http://dx.doi.org/10.1016/S1470-2045(21)00344-2
21. Gorin MA, Rowe SP, Allaf ME. Oncocytic Neoplasm on Renal Mass Biopsy: A Diagnostic Conundrum. Oncology (Williston Park). 2016 May;30(5):426–35.

For peer review only - http://bmjopen.bmj.com/site/about/guidelines.xhtml

22. Vindrola-Padros C, Chisnall G, Cooper S, Dowrick A, Djellouli N, Symmons SM, et al. Carrying Out Rapid Qualitative Research During a Pandemic: Emerging Lessons From COVID-19. Qualitative Health Research. 2020;30(14):2192–204.
23. Gale NK, Heath G, Cameron E, Rashid S, Redwood S. Using the framework method for the analysis of qualitative data in multi-disciplinary health research. BMC Medical Research Methodology. 2013;13(117).
24. Gorin MA, Rowe SP, Baras AS, Solnes LB, Ball MW, Pierorazio PM, et al. Prospective Evaluation of 99mTc-sestamibi SPECT/CT for the Diagnosis of Renal Oncocytomas and Hybrid Oncocytic/Chromophobe Tumors. European Urology. 2015;69(3):413–6.
25. Shinkins B, Thompson M, Mallett S, Perera R. Diagnostic accuracy studies : how to report and analyse. British Medical Journal. 2013;2778(May):1–11.
26. Gwet K. Handbook of inter-rater reliability. The definitive guide to measuring the extent of agreement amongst raters. 4th Editio. Gaithersburg M 20886 2696, editor. Advances Analytics, LLC; 2014.
27. Love-Koh J. How Useful Are Early Economic Models? Comment on “Problems and Promises of Health Technologies: The Role of Early Health Economic Modelling.” Int J Health Policy Manag. 2020;9(5):215–7.
28. Su ZT, Patel HD, Huang MM, Meyer AR, Pavlovich CP, Pierorazio PM, et al. Cost-effectiveness Analysis of 99mTc-sestamibi SPECT/CT to Guide Management of Small Renal Masses. European Urology Focus. 2021;7(4):827–34.
29. Sperber D, et al. An expert on every street corner? Methods for eliciting distributions in geographically dispersed opinion pools. Value Health. 2013;16(2):434–7.
30. Janssen A, Pickard A, Golicki D, Gudex C, Niewada M, Scalone L, et al. Measurement properties of the EQ-5D-5L compared to the EQ-5D-3L across eight patient groups: a multi-country study. Qual Life Res. 2013;22(7):1717–27.
31. UK Health Security Agency. Ionising radiation: dose comparisons [Internet]. Guidance Document. 2011 [cited 2022 May 24]. Available from: https://www.gov.uk/government/publications/ionising-radiation-dose-comparisons/ionising-radiation-dose-comparisons

Figure 1: Study flow diagram
Table 1: Visit schedule and assessments

For peer review only

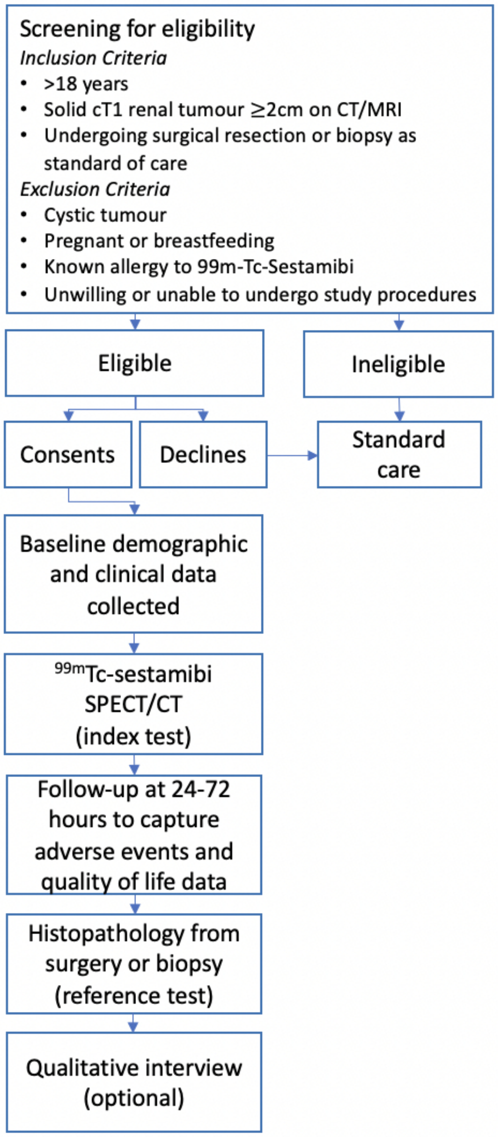

Figure 1: Study flow diagram

92x196mm (144 x 144 DPI)

STANDARD PROTOCOL ITEMS: RECOMMENDATIONS FOR INTERVENTIONAL TRIALS

SPIRIT 2013 Checklist: Recommended items to address in a clinical trial protocol and related documents*

Section/item	Item No	Description
**Administrative information**		
Title	1	Descriptive title identifying the study design, population, interventions, and, if applicable, trial acronym (page 4, line 2)
Trial registration	2a	Trial identifier and registry name. If not yet registered, name of intended registry (page 5, line 41)
	2b	All items from the World Health Organization Trial Registration Data Set (throughout text)
Protocol version	3	Date and version identifier (page 5, line 39)
Funding	4	Sources and types of financial, material, and other support (page 14, line 18)
Roles and responsibilities	5a	Names, affiliations, and roles of protocol contributors (page 4, line 9)
	5b	Name and contact information for the trial sponsor (page 12, line 40)
	5c	Role of study sponsor and funders, if any, in study design; collection, management, analysis, and interpretation of data; writing of the report; and the decision to submit the report for publication, including whether they will have ultimate authority over any of these activities (page 14, line 3-21)
	5d	Composition, roles, and responsibilities of the coordinating centre, steering committee, endpoint adjudication committee, data management team, and other individuals or groups overseeing the trial, if applicable (see Item 21a for data monitoring committee) (page 14, line 3-21)
**Introduction**		
Background and rationale	6a	Description of research question and justification for undertaking the trial, including summary of relevant studies (published and unpublished) examining benefits and harms for each intervention (page 6, from line 3)
	6b	Explanation for choice of comparators (page 8, line 33)
Objectives	7	Specific objectives or hypotheses (page 7, line 25)

For peer review only - http://bmjopen.bmj.com/site/about/guidelines.xhtml

Trial design	8	Description of trial design including type of trial (eg, parallel group, crossover, factorial, single group), allocation ratio, and framework (eg, superiority, equivalence, noninferiority, exploratory) (page 7, line 18)
**Methods: Participants, interventions, and outcomes**		
Study setting	9	Description of study settings (eg, community clinic, academic hospital) and list of countries where data will be collected. Reference to where list of study sites can be obtained (page 8, line 13)
Eligibility criteria	10	Inclusion and exclusion criteria for participants. If applicable, eligibility criteria for study centres and individuals who will perform the interventions (eg, surgeons, psychotherapists) (page 8, line 17)
Interventions	11a	Interventions for each group with sufficient detail to allow replication, including how and when they will be administered (page 8, line 34)
	11b	Criteria for discontinuing or modifying allocated interventions for a given trial participant (eg, drug dose change in response to harms, participant request, or improving/worsening disease) N/A
	11c	Strategies to improve adherence to intervention protocols, and any procedures for monitoring adherence (eg, drug tablet return, laboratory tests) N/A
	11d	Relevant concomitant care and interventions that are permitted or prohibited during the trial N/A
Outcomes	12	Primary, secondary, and other outcomes, including the specific measurement variable (eg, systolic blood pressure), analysis metric (eg, change from baseline, final value, time to event), method of aggregation (eg, median, proportion), and time point for each outcome. Explanation of the clinical relevance of chosen efficacy and harm outcomes is strongly recommended (page 7, line 54)
Participant timeline	13	Time schedule of enrolment, interventions (including any run-ins and washouts), assessments, and visits for participants. A schematic diagram is highly recommended (see Figure) (page 13, table)
Sample size	14	Estimated number of participants needed to achieve study objectives and how it was determined, including clinical and statistical assumptions supporting any sample size calculations (page 9, line 34)
Recruitment	15	Strategies for achieving adequate participant enrolment to reach target sample size (page 9, line 54)
**Methods: Assignment of interventions (for controlled trials) N/A**		
Allocation:		

Sequence generation	16a	Method of generating the allocation sequence (eg, computer-generated random numbers), and list of any factors for stratification. To reduce predictability of a random sequence, details of any planned restriction (eg, blocking) should be provided in a separate document that is unavailable to those who enrol participants or assign interventions N/A
Allocation concealment mechanism	16b	Mechanism of implementing the allocation sequence (eg, central telephone; sequentially numbered, opaque, sealed envelopes), describing any steps to conceal the sequence until interventions are assigned N/A
Implementation	16c	Who will generate the allocation sequence, who will enrol participants, and who will assign participants to interventions N/A
Blinding (masking)	17a	Who will be blinded after assignment to interventions (eg, trial participants, care providers, outcome assessors, data analysts), and how N/A
	17b	If blinded, circumstances under which unblinding is permissible, and procedure for revealing a participant's allocated intervention during the trial N/A
**Methods: Data collection, management, and analysis**		
Data collection methods	18a	Plans for assessment and collection of outcome, baseline, and other trial data, including any related processes to promote data quality (eg, duplicate measurements, training of assessors) and a description of study instruments (eg, questionnaires, laboratory tests) along with their reliability and validity, if known. Reference to where data collection forms can be found, if not in the protocol (page 13, table)
	18b	Plans to promote participant retention and complete follow-up, including list of any outcome data to be collected for participants who discontinue or deviate from intervention protocols (page 9, line 53)
Data management	19	Plans for data entry, coding, security, and storage, including any related processes to promote data quality (eg, double data entry; range checks for data values). Reference to where details of data management procedures can be found, if not in the protocol (page 11, line 31)
Statistical methods	20a	Statistical methods for analysing primary and secondary outcomes. Reference to where other details of the statistical analysis plan can be found, if not in the protocol (page 9, line 43 and page 10, line 19)
	20b	Methods for any additional analyses (eg, subgroup and adjusted analyses) N/A
	20c	Definition of analysis population relating to protocol non-adherence (eg, as randomised analysis), and any statistical methods to handle missing data (eg, multiple imputation) N/A

Methods: Monitoring

Data monitoring	21a	Composition of data monitoring committee (DMC); summary of its role and reporting structure; statement of whether it is independent from the sponsor and competing interests; and reference to where further details about its charter can be found, if not in the protocol. Alternatively, an explanation of why a DMC is not needed (page 12, line 34)
	21b	Description of any interim analyses and stopping guidelines, including who will have access to these interim results and make the final decision to terminate the trial N/A
Harms	22	Plans for collecting, assessing, reporting, and managing solicited and spontaneously reported adverse events and other unintended effects of trial interventions or trial conduct (page 12, line 37)
Auditing	23	Frequency and procedures for auditing trial conduct, if any, and whether the process will be independent from investigators and the sponsor (page 12, line 48)

Ethics and dissemination

Research ethics approval	24	Plans for seeking research ethics committee/institutional review board (REC/IRB) approval (page 12, line 53)
Protocol amendments	25	Plans for communicating important protocol modifications (eg, changes to eligibility criteria, outcomes, analyses) to relevant parties (eg, investigators, REC/IRBs, trial participants, trial registries, journals, regulators) (page 12, line 54)
Consent or assent	26a	Who will obtain informed consent or assent from potential trial participants or authorised surrogates, and how (see Item 32) (page 13, table)
	26b	Additional consent provisions for collection and use of participant data and biological specimens in ancillary studies, if applicable N/A
Confidentiality	27	How personal information about potential and enrolled participants will be collected, shared, and maintained in order to protect confidentiality before, during, and after the trial (page 11, line 32)
Declaration of interests	28	Financial and other competing interests for principal investigators for the overall trial and each study site (page 14, line 23)
Access to data	29	Statement of who will have access to the final trial dataset, and disclosure of contractual agreements that limit such access for investigators (page 11, line 43)
Ancillary and post-trial care	30	Provisions, if any, for ancillary and post-trial care, and for compensation to those who suffer harm from trial participation N/A

For peer review only

For peer review only - http://bmjopen.bmj.com/site/about/guidelines.xhtml

Dissemination policy	31a	Plans for investigators and sponsor to communicate trial results to participants, healthcare professionals, the public, and other relevant groups (eg, via publication, reporting in results databases, or other data sharing arrangements), including any publication restrictions (page 13, line 4)
	31b	Authorship eligibility guidelines and any intended use of professional writers N/A
	31c	Plans, if any, for granting public access to the full protocol, participant-level dataset, and statistical code N/A – this is the study protocol, being published in open access format
**Appendices**		
Informed consent materials	32	Model consent form and other related documentation given to participants and authorised surrogates Appendix
Biological specimens	33	Plans for collection, laboratory evaluation, and storage of biological specimens for genetic or molecular analysis in the current trial and for future use in ancillary studies, if applicable N/A

*It is strongly recommended that this checklist be read in conjunction with the SPIRIT 2013 Explanation & Elaboration for important clarification on the items. Amendments to the protocol should be tracked and dated. The SPIRIT checklist is copyrighted by the SPIRIT Group under the Creative Commons "Attribution-NonCommercial-NoDerivs 3.0 Unported" license.

For peer review only
