# Supplementary material for: Protocol for a MULTI-centre feasibility study to assess the use of 99mTc-sestaMIBI SPECT/CT in the diagnosis of kidney tumours (MULTI-MIBI study)
Source: BMJ Open. 2023 Jan 24;13(1):e067496. doi: 10.1136/bmjopen-2022-067496 (PMC9884914; doi:10.1136/bmjopen-2022-067496)
Supplement: Reviewer comments [file bmjopen-2022-067496.reviewer_comments.pdf]

PEER REVIEW HISTORY

BMJ Open publishes all reviews undertaken for accepted manuscripts. Reviewers are asked to complete a checklist review form (**http://bmjopen.bmj.com/site/about/resources/checklist.pdf**) and are provided with free text boxes to elaborate on their assessment. These free text comments are reproduced below.

ARTICLE DETAILS

**TITLE (PROVISIONAL)**	Protocol for a MULTI-centre feasibility study to assess the use of 99m Tc-sestaMIBI SPECT/CT in the diagnosis of kidney tumours (MULTI-MIBI study)
**AUTHORS**	Warren, Hannah; Wagner, Thomas; Gorin, Michael; Rowe, Steven; Holman, Beverley; Pencharz, Deborah; El-Sheikh, Soha; Barod, Ravi; Patki, Prasad; Mumtaz, Faiz; Bex, Axel; Kasivisvanathan, Veeru; Moore, Caroline; Campain, Nicholas; Cartledge, Jon; Scarsbrook, Andrew; Hassan, Fahim; O'Brien, Tim; Stewart, Grant; Mendichovszky, Iosif; Dizdarevic, Sabina; Alanbuki, Ammar; Wildgoose, William; Wah, Tze; Vindrola-Padros, Cecilia; Pizzo, Elena; Dehbi, Hakim-Moulay; Lorgelly, Paula; Gurusamy, Kurinchi; Emberton, Mark; Tran, Maxine

This article was not externally reviewed at BMJ Open. Protocols that have been independently assessed prior to submission to BMJ Open are usually fast-tracked to publication on the grounds that further substantial changes will not be possible. This independent assessment will usually be external, independent review for both a substantial grant award from a non-commercial or government funder and ethics approval.
